# Protection From Natural Immunity Against Enteric Infections and Etiology-Specific Diarrhea in a Longitudinal Birth Cohort

**DOI:** 10.1093/infdis/jiaa031

**Published:** 2020-01-27

**Authors:** Elizabeth T Rogawski McQuade, Jie Liu, Gagandeep Kang, Margaret N Kosek, Aldo A M Lima, Pascal O Bessong, Amidou Samie, Rashidul Haque, Estomih R Mduma, Sanjaya Shrestha, Jose Paulo Leite, Ladaporn Bodhidatta, Najeeha Iqbal, Nicola Page, Ireen Kiwelu, Zulfiqar Bhutta, Tahmeed Ahmed, Eric R Houpt, James A Platts-Mills

**Affiliations:** 1 Department of Public Health Sciences, University of Virginia, Charlottesville, Virgina, USA; 2 Division of Infectious Diseases & International Health, University of Virginia, Charlottesville, Virgina, USA; 3 Christian Medical College, Vellore, India; 4 Asociación Benéfica PRISMA, Iquitos, Peru; 5 Federal University of Ceara, Fortaleza, Brazil; 6 University of Venda, Thohoyandou, South Africa; 7 International Centre for Diarrheal Disease Research, Dhaka, Bangladesh; 8 Haydom Global Health Research Centre, Haydom, Tanzania; 9 Walter Reed/AFRIMS Research Unit, Kathmandu, Nepal; 10 Fundação Oswaldo Cruz (Fiocruz), Rio de Janeiro, Brazil; 11 Armed Forces Research Institute of Medical Sciences (AFRIMS), Bangkok, Thailand; 12 Aga Khan University, Karachi, Pakistan; 13 National Institute for Communicable Diseases, Johannesburg, South Africa; 14 Kilimanjaro Clinical Research Institute, Moshi, Tanzania

**Keywords:** bias analysis, diarrhea, enteric infections, natural immunity, negative control

## Abstract

**Background:**

The degree of protection conferred by natural immunity is unknown for many enteropathogens, but it is important to support the development of enteric vaccines.

**Methods:**

We used the Andersen-Gill extension of the Cox model to estimate the effects of previous infections on the incidence of subsequent subclinical infections and diarrhea in children under 2 using quantitative molecular diagnostics in the MAL-ED cohort. We used cross-pathogen negative control associations to correct bias due to confounding by unmeasured heterogeneity of exposure and susceptibility.

**Results:**

Prior rotavirus infection was associated with a 50% lower hazard (calibrated hazard ratio [cHR], 0.50; 95% confidence interval [CI], 0.41–0.62) of subsequent rotavirus diarrhea. Strong protection was evident against *Cryptosporidium* diarrhea (cHR, 0.32; 95% CI, 0.20–0.51). There was also protection due to prior infections for norovirus GII (cHR against diarrhea, 0.67; 95% CI, 0.49–0.91), astrovirus (cHR, 0.62; 95% CI, 0.48–0.81), and *Shigella* (cHR, 0.79; 95% CI, 0.65–0.95). Minimal protection was observed for other bacteria, adenovirus 40/41, and sapovirus.

**Conclusions:**

Natural immunity was generally stronger for the enteric viruses than bacteria, potentially due to less antigenic diversity. Vaccines against major causes of diarrhea may be feasible but likely need to be more immunogenic than natural infection.

## Teaser

We estimated natural immunity to enteric pathogens as the effects of previous infections on the incidence of subsequent infections in children under 2. Prior rotavirus, *Cryptosporidium*, norovirus GII, astrovirus, and *Shigella* infections were associated with lower risk of subsequent diarrhea.

**(See the Editorial Commentary by Lopman and Baker, on pages 1764–7.)**

Vaccines are currently under development for several leading causes of diarrhea among children in low-resource settings, including *Shigella* [1], enterotoxigenic Escherichia *coli* [2], norovirus [3], and *Campylobacter* [4]. The development of immunity after natural infection is an important guide towards a successful vaccine. For rotavirus, observational analyses of natural immunity in low-resource settings found levels of protection that were comparable to that of vaccine efficacy [5–7], suggesting that natural immunity was a good predictor of vaccine performance. Assessment of natural immunity has informed cholera vaccine development, and levels of natural immunity are frequently used as a benchmark against which to judge vaccine candidates [8, 9]. However, for many of the highest burden diarrhea etiologies, the degree of protection conferred by natural immunity is unknown, and immunologic surrogates of protection are imperfect. As large-scale investments in new vaccines are being made, better understanding of the magnitude, if any, of natural immunity to enteric pathogens is needed to inform expectations for vaccine effectiveness.

Estimates of natural immunity are confounded by the fact that children who are infected with an enteric pathogen may be more likely to be infected again due to high exposure risk and/or greater host susceptibility compared with other children. For example, children with a water source that is contaminated with *Cryptosporidium* will be more likely than other children to be infected again, even if they acquire some immunity after their first infection. Because this bias is expected to be in the opposite direction of a protective effect of natural immunity, confounding may completely mask evidence of protection or identify prior infection as a risk factor for subsequent infection.

Although observed factors such as sociodemographics, environmental characteristics, and markers of malnutrition may be able to explain some of this heterogeneity, these variables have been only modestly associated with acquisition of specific enteric infections among children in low-resource settings [10–14], suggesting that it is difficult to predict exposure and susceptibility based on readily observed characteristics. Unmeasured factors, such as innate immunity (major histocompatibility complex variation), are likely also important to characterize individual susceptibility, but they are difficult to account for in this setting. Alternatively, exposure to other enteropathogens can be used as negative controls because prior exposure to other pathogens would not be expected to elicit immunity, but it may be a good proxy for exposure and susceptibility due to common transmission pathways and host-related risk factors.

In this study, we estimated the protective effects of natural infection by common enteric pathogens against subsequent subclinical infection and etiology-specific diarrhea identified by quantitative molecular diagnostics in a longitudinal birth cohort. We used cross-pathogen estimates of protection as negative controls to estimate the magnitude of cofounding bias due unmeasured heterogeneity of exposure and susceptibility and calibrate effect estimates to account for this systematic error.

## METHODS

The study design and methods of the MAL-ED study have been previously described [15]. In brief, children were enrolled within 17 days of birth from November 2009 to February 2012 at 8 sites: Dhaka, Bangladesh; Fortaleza, Brazil; Vellore, India; Bhaktapur, Nepal; Naushahro Feroze, Pakistan; Loreto, Peru; Venda, South Africa; and Haydom, Tanzania. Each site obtained ethical approval from their institutions, and written informed consent was obtained from participants. Children were excluded if their mother was <16 years of age, their family intended to move from the study area, they were from a multiple pregnancy, their birthweight was ≤1500 grams, or they were diagnosed with congenital or severe neonatal disease. Surveillance was conducted for diarrhea, defined as maternal report of ≥3 loose stools in 24 hours or 1 stool with visible blood, at twice-weekly home visits until 2 years of age. Stool samples were collected monthly and during diarrhea episodes.

Stool samples from the subset of children with complete follow-up to 2 years of age were tested for 29 enteropathogens (Supplementary Table S1) by quantitative polymerase chain reaction (qPCR) using custom-designed TaqMan Array Cards (Thermo Fisher Scientific, Carlsbad, CA). Details of the assays and quality control have been previously described [16, 17]. Pathogen-attributable diarrhea episodes were defined using adjusted attributable fractions (AFes) for each episode to account for subclinical infections [16, 18]. In brief, we used the pathogen quantity, age, and sex-specific odds ratios (ORs) for diarrhea to estimate AFes for each diarrhea episode as follows: 1 – 1/OR. We defined pathogen-attributable episodes when the pathogen quantity-derived AFe was .5 or higher (ie, majority attribution), as previously described [19]. In a sensitivity analysis, we excluded diarrhea episodes in which more than 1 etiology was identified. Severe pathogen-attributable diarrhea episodes were defined by a severity score greater than 6, derived from components of the Vesikari score [20].

Because low-quantity detection of pathogen nucleic acid by qPCR may not indicate an established infection, we defined infections that could confer natural immunity as any detection at a quantity corresponding to qPCR cycle threshold (Cq) value ≤30. For pathogens in which lower quantities were associated with diarrhea (where AFe ≥.5), the quantity associated with diarrhea was used (rotavirus, Cq ≤32.638; *Shigella*, Cq ≤30.507; adenovirus 40/41, Cq ≤30.424; and norovirus GII, Cq ≤30.357). In sensitivity analyses, we (1) used the more sensitive analytical cutoff of Cq <35 to define infections and (2) considered only attributable diarrhea episodes as able to confer natural immunity, a more specific definition. We further limited to new infections that occurred at least 21 days after a previous detection. In sensitivity analyses, we defined new infections after 14 and 31 days.

### Data Analysis

To estimate protection due to natural immunity, we estimated the effects of prior infections on the hazard of subsequent subclinical infections, diarrhea, and severe diarrhea episodes due to the same pathogen. We assessed natural immunity to the 10 most common causes of diarrhea in MAL-ED: rotavirus, adenovirus 40/41, norovirus GII, sapovirus, astrovirus, *Shigella*, *Campylobacter jejuni/Campylobacter coli*, heat-stable enterotoxigenic *E coli* (ST-ETEC), typical enteropathogenic *E coli* (tEPEC), and *Cryptosporidium* [18]. We also assessed protection against subclinical infection for enteric pathogens with prevalence ≥.5%: norovirus GI, enteroaggregative *E coli*, atypical EPEC, heat-labile enterotoxigenic *E coli* (LT-ETEC), *Aeromonas*, Helicobacter *pylori*, *Plesiomonas*, STEC, *Salmonella*, *Giardia*, *Enterocytozoon bieneusi*, and *Cyclospora.*

To model these effects, we used the Andersen and Gill extension of the Cox model for recurrent events [21] with a counting process formulation. Each risk period was defined by birth or age at 21 days after a prior infection to age at subsequent outcome, and each child contributed multiple risk periods from birth to 2 years of age (Supplementary Figure S1). Therefore, baseline hazards by age were estimated non-parametrically, and hazards of subsequent outcomes were conditioned on age. We estimated protection due to natural immunity as follows: (1-hazard ratio [HR]) × 100, in which the HR compared children who had experienced 1 or 2+ prior infections to children who experienced no prior infections. Robust variance accounted for correlation between risk periods within each child [22]. Models were adjusted for site and prespecified risk factors for enteric infections identified in previous analyses of MAL-ED [10–14]: sex, socioeconomic status (WAMI index [23]), enrollment weight-for-age z-score, maternal height, maternal education, crowding in the home, and percentage of days exclusively breastfed (from birth to the current risk period up to 6 months of age). In a sensitivity analysis, we stratified effects by age.

### Bias Calibration

We used cross-pathogen estimates of protection, that is, associations between each enteric infection outcome and prior exposure to a different pathogen (eg, the protection due to prior *Campylobacter* infection on subsequent *Shigella* diarrhea) as negative control associations. We considered pathogens of the same type (bacteria, viruses, and parasites) as negative controls for each of the enteric infections. Because there were only 4 parasites, both bacteria and parasites were included as negative controls for *Cryptosporidium*. In sensitivity analyses, we included all pathogens as negative controls and compared calibrated estimates to estimates additionally adjusted for prior exposure to other pathogens.

We calibrated estimates with the negative controls using methods previously described [24, 25] with the EmpiricalCalibration package in R. After estimating the negative control associations using the models above, we used maximum likelihood to generate a systematic error model for each pathogen outcome that fit a Gaussian distribution to the negative control estimates and accounted for the sampling error of each estimate. Assuming the systematic error does not change as a function of the true effect size, we then estimated a calibrated distribution for the effect of interest that incorporated both random error and the systematic error model estimated above. We computed calibrated effect estimates and confidence intervals (CIs) as the .5, .025, and .975 percentiles of the corresponding cumulative distribution function [25].

## RESULTS

Among 1715 children with follow-up to 2 years and molecular testing of stool samples, there were a total of 52 382 detections of the top 10 causes of diarrhea (Table 1). Half of these detections (n = 24 520, 46.8%) were at quantities of Cq ≤30 (or greater than the diarrhea-associated quantity) and 21 days distant from a prior infection. Among these infections, the children experienced 3526 attributable diarrhea episodes and 539 severe attributable diarrhea episodes. For each pathogen, more than half of children had at least 1 infection, except for rotavirus (48.9% of children). *Shigella* (n = 719 episodes; 29.6% of children with 1+ episodes) and rotavirus (n = 552; 26.0%) were the most common causes of diarrhea (Table 1).

**Table 1.** Number of Etiology-Specific Infections, Attributable Diarrhea Episodes, and Severe Attributable Diarrhea Episodes Among 1715 Children in the MAL-ED Cohort

Number (%)	Rotavirus	Astrovirus	Norovirus GII	Sapovirus	Adenovirus 40/41	*Shigella*	ST-ETEC	tEPEC	*Campylobacter jejuni/ Campylobacter coli*	*Cryptosporidium*
Detections (Cq <35)	2239	5037	5526	6094	6112	4744	6314	5197	7734	3385
Infections (Cq ≤30^a^)										
Total	1307 (58.4)	1886 (37.4)	2951 (53.4)	3043 (49.9)	2443 (40.0)	2901 (61.2)	3343 (52.9)	3041 (58.5)	3860 (49.9)	1625 (48.0)
New^b^	1217 (93.1)	1759 (93.3)	2717 (92.1)	2801 (92.0)	2264 (92.7)	2648 (91.3)	3216 (96.2)	2849 (93.7)	3596 (93.2)	1453 (89.4)
Mean age (days; SD)	339 (188.0)	388 (191.5)	340 (174.9)	419 (172.7)	398 (190.5)	508 (159.2)	435 (178.5)	381 (183.6)	391 (178.2)	467 (164.0)
Children with 1	568 (33.1)	566 (33.0)	511 (29.8)	517 (30.1)	570 (33.2)	422 (24.6)	419 (24.4)	416 (24.3)	341 (19.9)	603 (35.2)
Children with 2+	271 (15.8)	482 (28.1)	799 (46.6)	833 (48.6)	591 (34.5)	719 (41.9)	825 (48.1)	835 (48.7)	850 (49.6)	347 (20.2)
Attributable Diarrhea Episodes										
Total	566	308	296	542	411	755	452	31	162	119
New^b^	552 (97.5)	302 (98.1)	293 (99.0)	526 (97.0)	390 (94.9)	719 (95.2)	446 (98.7)	31 (100)	156 (96.3)	111 (93.3)
Mean age (days; SD)	348 (170.1)	396 (177.7)	377 (160.5)	428 (150.2)	357 (169.3)	507 (152.0)	442 (162.9)	223 (144.3)	338 (160.1)	478 (170.1)
Children with 1	353 (20.6)	224 (13.1)	218 (12.7)	276 (16.1)	157 (9.2)	347 (20.2)	248 (14.5)	27 (1.6)	99 (5.8)	93 (5.4)
Children with 2+	93 (5.4)	37 (2.2)	36 (2.1)	112 (6.5)	90 (5.2)	160 (9.3)	82 (4.8)	2 (0.1)	25 (1.5)	9 (0.5)
Severe Attributable Diarrhea Episodes										
Total	156	46	40	67	61	90	62	8	12	16
New^b^	152 (97.4)	46 (100)	40 (100)	65 (97.0)	56 (91.8)	87 (96.7)	61 (98.4)	8 (100)	10 (83.3)	14 (87.5)
Mean age (days; SD)	302 (144.4)	306 (149.5)	389 (150.9)	413 (150.6)	313 (143.2)	459 (165.3)	386 (167.8)	204 (136.7)	364 (112.5)	384 (175.7)
Children with 1	137 (8.0)	46 (2.7)	38 (2.2)	52 (3.0)	49 (2.9)	77 (4.5)	59 (3.4)	8 (0.5)	10 (0.6)	14 (0.8)
Children with 2+	7 (0.4)	0	1 (0.1)	6 (0.4)	3 (0.2)	5 (0.3)	1 (0.1)	0	0	0

Abbreviations: Cq, quantitative polymerase chain reaction cycle threshold; SD, standard deviation.

^a^For pathogens in which lower quantities were associated with diarrhea (where adjusted attributable fraction ≥.5), the quantity associated with diarrhea was used to define infections (rotavirus, Cq ≤32.638; *Shigella*, Cq ≤30.507; adenovirus 40/41, Cq ≤30.424; and norovirus GII, Cq ≤30.357).

^b^Infections and episodes were considered new and included in the analysis if they occurred at least 21 days distant from a previous detection.

**Figure 1.**
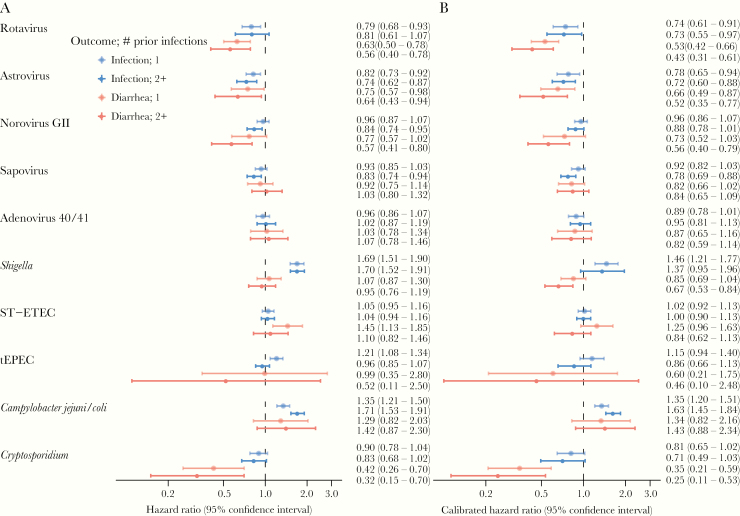
Estimates of protection against subclinical infection and attributable diarrhea due to 1 or 2 or more prior infections (subclinical or diarrhea) from the same pathogen in the MAL-ED cohort. (A) Hazard ratios adjusted for site, socioeconomic status, sex, enrollment weight-for-age Z-score, maternal education, maternal height, crowding, and exclusive breastfeeding in first 6 months; (B) hazard ratios adjusted for the same variables and calibrated based on negative control estimates. Abbreviations: ST-ETEC, heat-stable enterotoxigenic E coli; tEPEC, typical enteropathogenic E coli.

Covariate-adjusted estimates of protection against subsequent subclinical infection and attributable diarrhea due to prior infection (subclinical or diarrhea) (Figure 1A) were nearly equivalent to the corresponding unadjusted estimates (Supplementary Table S2). However, cross-pathogen negative control associations generated systematic bias distributions that were above the null for almost all pathogen outcomes, suggesting that the adjusted estimates were biased upwards and underestimated natural immunity (Supplementary Figure S2). The magnitude of estimated bias (Table 2) was generally larger for the diarrhea outcomes than the infection outcomes, and it was larger for estimates assessing 2+ prior infections compared with 1 prior infection. *Shigella* demonstrated the largest magnitude of bias. Hazard ratios between *Shigella* diarrhea and 2+ prior infections by negative control pathogens were systematically 42% higher than the expected null association (mean adjusted HR for systematic error, 1.42; 95% CI, 1.34–1.51). The viruses, rotavirus, astrovirus, sapovirus, and adenovirus 40/41 had similar bias distributions, with negative control associations an average of 18% higher than the null for the diarrhea outcomes. In contrast, there was no systematic error identified for norovirus GII or *C jejuni/C coli* (Table 2).

**Table 2.** Estimates of the Systematic Bias Distribution From Negative Control Cross-Pathogen Associations Between Pathogen Outcomes and Prior Exposure to Other Pathogens in the Same Group

Pathogen	Outcome	No. of Previous Infections	Mean Bias^a^	Bias Standard Deviation	Hazard Ratio^b^ (95% CI)
Rotavirus	Infection	1	0.06	0.07	1.07 (0.93–1.22)
		2+	0.10	0.03	1.10 (1.04–1.18)
	Diarrhea	1	0.17	0.02	1.18 (1.13–1.24)
		2+	0.26	0.05	1.30 (1.19–1.42)
Astrovirus	Infection	1	0.05	0.07	1.05 (0.91–1.21)
		2+	0.02	0.05	1.02 (0.93–1.12)
	Diarrhea	1	0.13	0.04	1.14 (1.06–1.22)
		2+	0.21	0.04	1.23 (1.14–1.33)
Norovirus GII	Infection	1	0.01	0.02	1.01 (0.98–1.04)
		2+	−0.05	0.02	0.95 (0.91–0.99)
	Diarrhea	1	0.05	0.10	1.05 (0.87–1.27)
		2+	0.02	0.04	1.02 (0.95–1.09)
Sapovirus	Infection	1	0.02	0.03	1.02 (0.96–1.08)
		2+	0.07	0.02	1.07 (1.03–1.11)
	Diarrhea	1	0.11	0.02	1.12 (1.07–1.17)
		2+	0.20	0.03	1.22 (1.14–1.31)
Adenovirus 40/41	Infection	1	0.08	0.03	1.08 (1.02–1.15)
		2+	0.07	0.03	1.07 (1.00–1.14)
	Diarrhea	1	0.17	0.05	1.18 (1.07–1.30)
		2+	0.26	0.05	1.30 (1.18–1.43)
*Shigella*	Infection	1	0.15	0.08	1.16 (0.99–1.35)
		2+	0.22	0.17	1.25 (0.89–1.75)
	Diarrhea	1	0.23	0.02	1.26 (1.20–1.32)
		2+	0.35	0.03	1.42 (1.34–1.51)
ST-ETEC	Infection	1	0.03	0.02	1.03 (0.99–1.07)
		2+	0.04	0.02	1.04 (1.00–1.08)
	Diarrhea	1	0.15	0.05	1.16 (1.05–1.28)
		2+	0.27	0.05	1.31 (1.20–1.43)
tEPEC	Infection	1	0.05	0.09	1.05 (0.89–1.24)
		2+	0.10	0.13	1.11 (0.87–1.42)
	Diarrhea	1	0.49	0.12	1.64 (1.30–2.06)
		2+	0.12	0.31	1.13 (0.61–2.07)
*Campylobacter jejuni/Campylobacter coli*	Infection	1	0.00	0.02	1.00 (0.96–1.04)
		2+	0.05	0.02	1.05 (1.00–1.10)
	Diarrhea	1	−0.03	0.09	0.97 (0.82–1.14)
		2+	−0.01	0.04	0.99 (0.92–1.07)
*Cryptosporidium*	Infection	1	0.10	0.09	1.10 (0.93–1.31)
		2+	0.15	0.15	1.17 (0.86–1.58)
	Diarrhea	1	0.20	0.06	1.22 (1.08–1.37)
		2+	0.28	0.05	1.32 (1.21–1.45)

Abbreviations: CI, confidence interval; ST-ETEC, heat-stable enterotoxigenic E coli; tEPEC, typical enteropathogenic E coli.

^a^Mean bias is estimated on the natural log scale.

^b^Hazard ratio = e^(mean bias)^.

After calibrating the adjusted estimates, estimates of protection generally increased (Figure 1B, Supplementary Table S2). One prior rotavirus infection was associated with an adjusted 26% lower hazard (calibrated HR [cHR], .74; 95% CI, .61–.91) of subsequent rotavirus infection and 47% lower hazard (cHR, .53; 95% CI, .42–.66) of subsequent rotavirus diarrhea (Figure 1A, Supplementary Table S2). Two or more prior rotavirus infections were associated with similar protection against infection but larger protection against diarrhea (57%; cHR, .43; 95% CI, .31–.61). There was slightly less protection for norovirus GII (cHR against diarrhea for 1+ prior infections, 0.67; 95% CI, 0.49–0.91) and astrovirus (cHR, 0.62; 95% CI, 0.48) (Supplementary Table S3).

The strongest protection was observed for *Cryptosporidium* diarrhea, and it was larger after multiple prior infections compared with 1 prior infection (cHR for 1 prior infection .0.35, 95% CI 0.21–0.59 vs cHR for 2+ prior infections 0.25, 95% CI 0.11–0.53). The calibrated estimates for *Shigella* diarrhea indicated modest protection after 1 prior infection (15%; cHR, .0.85; 95% CI, 0.69–1.04) and substantial protection after 2+ prior infections (33%; cHR, 0.67; 95% CI, 0.53–0.84). There was no evidence of protection after infections with the other bacterial pathogens (*C jejuni/C coli*, ETEC, tEPEC) and limited protection for adenovirus 40/41 and sapovirus (Figure 1B, Supplementary Table S2). Site-specific estimates of protection were generally consistent but were highly imprecise (Supplementary Table S4).

Estimates of protection against severe diarrhea were larger than those for episodes of any severity (Table 3). There was strong protection against severe diarrhea due to rotavirus (69%; cHR, 0.31; 95% CI, 0.20–0.48), astrovirus (66%; cHR, 0.34; 95% CI, 0.16–0.74), and *Cryptosporidium* (84%; cHR, 0.16; 95% CI, 0.02–1.72) after 1+ prior infections. Slightly less protection against severe *Shigella* diarrhea (43%, cHR, 0.57; 95% CI, 0.33–0.97) was also observed.

**Table 3.** Estimates of Protection Against Severe Attributable Diarrhea Episodes Due to 1 or More Prior Infections From the Same Pathogen in the MAL-ED Cohort

Pathogen	No. of Previous Infections	N	N (%) With Subsequent Severe Diarrhea	Unadjusted^a^ HR (95% CI)	Adjusted^b^ HR (95% CI)	Calibrated^c^ HR (95% CI)
Rotavirus	0	1715	121 (7.1)	1.	1.	1.
	1+	839	29 (3.5)	0.37 (0.24–0.58)	0.37 (0.24–0.58)	0.31 (0.20–0.48)
Astrovirus	0	1715	35 (2.0)	1.	1.	1.
	1+	1048	11 (1.0)	0.40 (0.19–0.85)	0.39 (0.18–0.84)	0.34 (0.16–0.74)
Norovirus GII	0	1715	15 (0.9)	1.	1.	1.
	1+	1310	24 (1.8)	0.70 (0.31–1.55)	0.76 (0.34–1.70)	0.73 (0.32–1.65)
Sapovirus	0	1715	27 (1.6)	1.	1.	1.
	1+	1350	34 (2.5)	1.12 (0.64–1.97)	1.11 (0.62–1.96)	0.97 (0.55–1.72)
Adenovirus 40/41	0	1715	29 (1.7)	1.	1.	1.
	1+	1161	26 (2.2)	0.91 (0.54–1.51)	0.89 (0.51–1.54)	0.74 (0.42–1.29)
*Shigella*	0	1715	62 (3.6)	1.	1.	1.
	1+	1141	24 (2.1)	0.78 (0.45–1.34)	0.73 (0.43–1.25)	0.57 (0.33–0.97)
ST-ETEC	0	1715	28 (1.6)	1.	1.	1.
	1+	1244	33 (2.7)	1.01 (0.53–1.89)	0.97 (0.52–1.83)	0.82 (0.43–1.56)
tEPEC	0	1715	8 (0.5)	—	—	—
	1+	1251	0	—	—	—
*Campylobacter jejuni/Campylobacter coli*	0	1715	3 (0.2)	1.	1.	1.
	1+	1191	7 (0.6)	1.82 (0.49–6.70)	1.71 (0.46–6.33)	1.81 (0.49–6.70)
*Cryptosporidium*	0	1715	13 (0.8)	1.	1.	1.
	1+	950	1 (0.1)	0.21 (0.02–2.15)	0.20 (0.02–2.11)	0.16 (0.02–1.72)

Abbreviations: CI, confidence interval; HR, hazard ratio; ST-ETEC, heat-stable enterotoxigenic E coli; tEPEC, typical enteropathogenic E coli.

^a^Estimates adjusted for site.

^b^Estimates adjusted for site, socioeconomic status, sex, enrollment weight-for-age z-score, maternal education, maternal height, crowding, and exclusive breastfeeding in first 6 months.

^c^Estimates adjusted for the same variables above and calibrated based on negative control estimates for the associations between 1 or more previous infections and attributable diarrhea of any severity.

There was no evidence of protection against bacteria or parasites that were infrequently associated with diarrhea (Table 4). In contrast, prior infection was strongly associated with subsequent infection for many, most strikingly for *Giardia*, *Salmonella*, *H pylori*, and *Cyclospora*. Estimates of natural protection against infection for norovirus GI were consistent with those for norovirus GII.

**Table 4.** Estimates of Protection Against Infection Due to 1 or 2 or More Prior Infections From the Same Pathogen for Enteric Infections That Were Not Associated With Diarrhea in the MAL-ED Cohort

Pathogen	No. of Previous Infections	N	N (%) With Subsequent Infection	Unadjusted^a^ HR (95% CI)	Adjusted^b^ HR (95% CI)	Calibrated^c^ HR (95% CI)
Norovirus GI	0	1715	622 (36.3)	1.	1.	1.
	1	622	143 (23.0)	0.92 (0.76–1.11)	0.88 (0.73–1.07)	0.90 (0.73–1.11)
	2+	143	18 (12.6)	0.57 (0.36–0.89)	0.53 (0.34–0.84)	0.51 (0.33–0.81)
EAEC	0	1715	1689 (98.5)	1.	1.	1.
	1	1689	1620 (95.9)	1.86 (1.72–2.01)	1.87 (1.73–2.02)	1.73 (1.57–1.90)
	2+	1620	1519 (93.8)	2.19 (2.03–2.35)	2.20 (2.04–2.37)	2.10 (1.92–2.30)
aEPEC	0	1715	1493 (87.1)	1.	1.	1.
	1	1493	1188 (79.6)	1.21 (1.11–1.32)	1.20 (1.10–1.31)	1.17 (1.06–1.29)
	2+	1188	862 (72.6)	1.34 (1.22–1.46)	1.31 (1.19–1.44)	1.22 (1.10–1.35)
LT-only ETEC	0	1715	1225 (71.4)	1.	1.	1.
	1	1225	758 (61.9)	1.18 (1.07–1.31)	1.18 (1.06–1.30)	1.37 (1.19–1.57)
	2+	758	406 (53.6)	1.17 (1.05–1.31)	1.17 (1.04–1.31)	1.25 (1.08–1.44)
*Aeromonas*	0	1715	90 (5.2)	1.	1.	1.
	1	90	9 (10.0)	3.31 (1.59–6.90)	3.01 (1.39–6.51)	2.87 (1.32–6.25)
	2+	9	1 (11.1)	3.46 (0.41–29.08)	2.63 (0.26–26.86)	3.12 (0.30–31.90)
*Helicobacter pylori*	0	1715	56 (3.3)	1.	1.	1.
	1	56	9 (16.1)	8.96 (3.75–21.43)	8.33 (3.45–20.14)	10.10 (4.15–24.53)
	2+	9	6 (66.7)	39.99 (19.32–82.78)	31.97 (18.61–54.93)	38.45 (21.79–67.83)
*Plesiomonas*	0	1715	170 (9.9)	1.	1.	1.
	1	170	37 (21.8)	2.54 (1.71–3.79)	2.43 (1.63–3.61)	2.64 (1.61–4.32)
	2+	37	8 (21.6)	2.77 (1.38–5.57)	2.49 (1.26–4.93)	2.62 (1.31–5.27)
STEC	0	1715	210 (12.2)	1.	1.	1.
	1	210	18 (8.6)	0.87 (0.53–1.44)	0.83 (0.50–1.40)	0.87 (0.50–1.53)
	2+	18	4 (22.2)	2.89 (1.05–7.97)	2.90 (1.01–8.34)	3.53 (1.22–10.19)
*Salmonella*	0	1715	66 (3.8)	1.	1.	1.
	1	66	5 (7.6)	3.36 (1.15–9.87)	3.11 (1.05–9.23)	2.86 (0.96–8.52)
	2+	5	3 (60.0)	25.23 (8.90–71.57)	19.20 (8.03–45.89)	16.07 (6.70–38.50)
*Giardia*	0	1715	1282 (74.8)	1.	1.	1.
	1	1282	1114 (86.9)	4.70 (4.19–5.29)	4.71 (4.19–5.29)	4.69 (4.01–5.49)
	2+	1114	1004 (90.1)	11.04 (10.13–12.04)	10.79 (9.89–11.78)	10.69 (8.21–13.92)
*Enterocytozoon bieneusi*	0	1715	723 (42.2)	1.	1.	1.
	1	723	304 (42.0)	1.99 (1.63–2.43)	1.94 (1.59–2.37)	1.93 (1.55–2.39)
	2+	304	91 (29.9)	1.80 (1.37–2.38)	1.73 (1.30–2.29)	1.68 (1.25–2.24)
*Cyclospora*	0	1715	110 (6.4)	1.	1.	1.
	1	110	49 (44.5)	11.14 (6.98–17.79)	11.91 (7.50–18.90)	8.59 (5.31–13.89)
	2+	49	20 (40.8)	6.70 (3.99–11.24)	6.09 (3.62–10.27)	4.13 (2.08–8.21)

Abbreviations: aEPEC, atypical enteropathogenic E coli; CI, confidence interval; EAEC, enteroaggregative E coli; HR, hazard ratio; LT-ETEC, heat-labile enterotoxigenic E coli; STEC, Shiga toxin-producing E coli.

^a^Estimates adjusted for site.

^b^Estimates adjusted for site, socioeconomic status, sex, enrollment weight-for-age z-score, maternal education, maternal height, crowding, and exclusive breastfeeding in first 6 months.

^c^Estimates adjusted for the same variables above and calibrated based on negative control estimates.

### Sensitivity Analyses

Greater protection in the second year of life compared with the first year of life was observed for adenovirus 40/41 and *Cryptosporidium* infection and tEPEC diarrhea. In contrast, prior sapovirus infection was protective against subsequent diarrhea in the first year but not the second (Supplementary Table S5).

Estimates of protection against rotavirus diarrhea were similar in the sites that had (Brazil, Peru, and South Africa) and had not introduced rotavirus vaccine. Protection against rotavirus infection was slightly stronger in sites that had introduced vaccine (cHR, 0.65; 95% CI, 0.42–1.00 for 1+ prior infection) compared with sites that had not (cHR, 0.76; 95% CI, 0.63–0.92).

Defining infections at a quantity cutoff of Cq <35 instead of Cq ≤30 resulted in inconsistent shifts in the estimates (Supplementary Table S6). For example, estimates of natural protection against diarrhea for rotavirus and astrovirus were closer to the null; estimates for norovirus GII and sapovirus were further from the null. Prior infections with *Cryptosporidium* were strongly predictive of subsequent infections, which may indicate that low-quantity detections are more likely to identify persistent infections rather than new infections (Supplementary Table S6).

Modification of the minimum duration between new infections (14 and 31 days instead of 21) resulted in minor changes to the estimates (Supplementary Tables S7 and S8). Defining prior exposure by prior attributable diarrhea instead of prior infection generally resulted in smaller estimates of protection, suggesting potential misclassification of immunity acquired in the absence of diarrheal symptoms (Supplementary Table S9). The exclusion of diarrhea episodes with multiple attributable pathogens resulted in slightly stronger estimates of protection for the enteric viruses (Supplementary Table S10). Including all pathogens as negative controls resulted in smaller estimates of bias, especially for the viruses, with calibrated estimates generally closer to the covariate-adjusted estimates (Supplementary Table S11). Finally, adjusting for prior exposure to other pathogens did not completely correct the bias identified by the negative control calibration approach (Supplementary Table S12).

## Discussion

We estimated strong protection due to prior infection against rotavirus, *Cryptosporidium*, and astrovirus diarrhea, which suggests that vaccine development for the latter 2 may be relatively feasible. Norovirus GII and *Shigella* also exhibited protection against diarrhea, but more strongly after multiple infections, which may reflect low potency or heterotypic protection. Less protection was observed for sapovirus and adenovirus 40/41. Estimates of protection against infection were universally smaller in magnitude, whereas estimates against severe diarrhea were larger, compared with diarrhea of any severity.

Observed levels of natural protection were generally consistent with previous literature. Estimates for rotavirus were similar to those in a previous study from India (40%–80% protection against diarrhea) [5], and they were smaller in magnitude to estimates from Mexico [6] and Guinea-Bissau [7] (70%–90%). These estimates were also consistent with estimates of rotavirus vaccine efficacy in low-resource settings [26], which supports the use of the models for other pathogens. Levels of natural protection against norovirus GII were consistent with previous analyses of MAL-ED using a subset of samples (25%–30% protection against diarrhea) [27] and smaller than estimates from Peru (50%–80%) [28]. In contrast, no natural immunity to norovirus was observed in a study in Ecuador [29]. Estimates for astrovirus were larger than those from previous analyses of MAL-ED using the enzyme immunoassay in a subset of samples [30] and those from a study in rural Egypt [31]. Protection for *Cryptosporidium* was larger against diarrhea (70% vs 25%) and smaller against subclinical infection (20% vs 50+%) compared with a study in India [32]. The evidence for natural immunity to *Cryptosporidium* is supported by protection observed among adults with pre-existing serum antibodies [33] and delayed cryptosporidiosis among children with higher levels of anti-*Cryptosporidium* fecal immunoglobulin A (IgA) [34].

Because many of the pathogens included in this analysis are immunologically heterogeneous, these results should be interpreted as estimates of “functional protection” that reflect both homotypic and heterotypic immunity at the population-level. Different degrees of pathogen heterogeneity may explain variations in levels of protection. *Shigella* is antigenically diverse with 4 species and more than 50 serotypes [35], such that poor cross-protection may explain the relatively limited observed natural protection. A previous analysis of infection-derived immunity to *Shigella* in Chile found 14% protection overall but more than 70% serotype-specific protection [36].

The lack of natural protection observed for sapovirus, ST-ETEC, and *C jejuni/C coli* may also be explained by antigenic diversity. Sapovirus has 4 genogroups and 16 genotypes [37], with a lack of cross-protection [38]. Protection against ETEC is conferred by immune responses to more than 20 different colonization factors [39, 40], and it was previously only observed for ETEC infections of the same toxin-colonization factor profile [41]. Likewise, protection against *Campylobacter* may be related to the polysaccharide capsule, which has a broad diversity of types [42].

The bias analysis using negative controls identified substantial unmeasured confounding in the covariate-adjusted estimates. Larger magnitudes of bias observed for the diarrhea than infection outcomes suggest that confounding by heterogeneity of susceptibility may be more important than heterogeneity of exposure because the latter would not be expected to differ based on the severity of the outcome. One source of heterogeneity in host susceptibility may be histoblood group antigens status [43]. It is interesting that bias was not observed for norovirus GII and *C jejuni/C coli*, which may reflect the tendency for norovirus to spread indiscriminately in populations (because it is a cause of outbreaks in other settings) [44] and the uniformly high frequency of *Campylobacter* detection [10, 19]. Persistent carriage of *Giardia* [11], *Salmonella* [45], and *H pylori* [46] may explain the strong positive associations with repeated detections for these pathogens, because subsequent detections may not be capturing new infections.

This analysis leverages previous work by using molecular diagnostics across pathogens and attributable fractions to adjudicate diarrhea etiology in the context of frequent subclinical infection. This study design allowed for the novel bias correction based on multiple negative control associations, which generated an empirical null distribution that captured a common distribution of biases [24], but did not require the structure and magnitude of confounding to be identical [47]. However, these analyses were limited by the inability to assess homotypic immunity. The poor sensitivities of typing assays applied directly to stool specimens for rotavirus, *Shigella*, and ST-ETEC resulted in more than 50% of detections being typed as other, which prohibited assessment of homotypic immunity. Furthermore, speciation and typing directly from stool rather than isolates limited our ability to ensure that (1) G and P types for rotavirus and (2) toxins and colonization factors for ETEC were detected in the same organism. In addition, with only monthly sampling, there was likely under-ascertainment of infections. In the 5 sites that had not introduced rotavirus vaccine, 30.4% (n = 238) of first rotavirus infections based on serologic testing at 7 and 15 months of age (IgA ≥20 U/mL) were not detected by qPCR. A total of 36.6% of these children were qPCR positive at an older age, at a median age delay of 5.0 months (interquartile range, 2.8–9.1). Because we were unable to make this comparison for the other pathogens, serology was not included as evidence of prior infection in the analysis.

## Conclusions

Estimates of natural protection for most enteric pathogens were modest and suggest that vaccines that simulate natural infections for the enteric bacteria are unlikely to provide important levels of protection. Vaccines currently in development, such as for *Shigella*, will likely need to provide heterotypic protection, be conjugated, and/or require boosting. Furthermore, protection against infection was limited, such that vaccines are unlikely to provide sterilizing immunity. Therefore, although vaccines could limit the acute burden of diarrheal illness and mortality, they may not effectively address the long-term impacts of subclinical infections, such as growth impairment [19].

## Supplementary Data

Supplementary materials are available at *The Journal of Infectious Diseases* online. Consisting of data provided by the authors to benefit the reader, the posted materials are not copyedited and are the sole responsibility of the authors, so questions or comments should be addressed to the corresponding author.

jiaa031_suppl_Supplementary_Material

## Notes

***Acknowledgments.*** We thank Martijn Schuemie and Laura Hester for their assistance with the methods for empirical calibration using negative controls. We also thank the staff and participants of the MAL-ED Network Project for their important contributions.

***Author contributions.*** E. T. R. M. conceived the study and led data analysis and writing of the report. J. L. led data acquisition and interpretation. G. K., M. N. K., A. A. M. L., P. O. B., A. S., R. H., E. R. M., S. S., J. P. L., L. B., N. I., N. P., I. K., Z. B., and T. A. contributed to site data acquisition, the design of the original protocol, and data interpretation. E. R. H. contributed to data acquisition, analysis, and interpretation. J. A. P.-M. conceived the study and contributed to data analysis and interpretation.

***Financial support.*** This work was funded by the National Institute of Allergy and Infectious Diseases at the National Institutes of Health (K01AI130326; to E. T. R. M.). The Etiology, Risk Factors and Interactions of Enteric Infections and Malnutrition and the Consequences for Child Health and Development Project (MAL-ED) was carried out as a collaborative project funded by the Bill & Melinda Gates Foundation (OPP1131125), the Foundation for the National Institutes of Health, and the Fogarty International Center.

***Potential conflicts of interest.*** All authors: No reported conflicts of interest. All authors have submitted the ICMJE Form for Disclosure of Potential Conflicts of Interest.
