# Supplementary material for: Protection From Natural Immunity Against Enteric Infections and Etiology-Specific Diarrhea in a Longitudinal Birth Cohort
Source: J Infect Dis. 2020 Jan 27;222(11):1858–68. doi: 10.1093/infdis/jiaa031 (PMC7653087; doi:10.1093/infdis/jiaa031)
Supplement: jiaa031_suppl_Supplementary_Material [file jiaa031_suppl_supplementary_material.pdf]

Supplementary material

Protection from natural immunity against enteric infections and etiology-specific diarrhea in a longitudinal birth cohort

Elizabeth T. Rogawski McQuade[1,2], Jie Liu[2], Gagandeep Kang[3], Margaret N. Kosek[2,4], Aldo A.M. Lima[5], Pascal O. Bessong[6], Amidou Samie[6], Rashidul Haque[7], Estomih R. Mduma[8], Sanjaya Shrestha[9], Jose Paulo Leite[10], Ladaporn Bodhidatta[11], Najeeha Iqbal[12], Nicola Page[13], Ireen Kiwelu[14], Zulfiqar Bhutta[12], Tahmeed Ahmed[7], Eric R. Houpt[2], James A. Platts-Mills[2]

[1]Department of Public Health Sciences, University of Virginia, Charlottesville, VA, USA
[2]Division of Infectious Diseases & International Health, University of Virginia, Charlottesville, VA, USA
[3]Christian Medical College, Vellore, India
[4]Asociación Benéfica PRISMA, Iquitos, Peru
[5]Federal University of Ceara, Fortaleza, Brazil
[6]University of Venda, Thohoyandou, South Africa
[7]International Centre for Diarrhoeal Disease Research, Bangladesh, Dhaka, Bangladesh
[8]Haydom Global Health Research Centre, Haydom, Tanzania
[9]Walter Reed/AFRIMS Research Unit, Nepal, Kathmandu, Nepal
[10]Fundação Oswaldo Cruz (Fiocruz), Rio de Janeiro, Brazil
[11]Armed Forces Research Institute of Medical Sciences (AFRIMS), Bangkok, Thailand
[12]Aga Khan University, Karachi, Pakistan
[13]National Institute for Communicable Diseases, Johannesburg, South Africa
[14]Kilimanjaro Clinical Research Institute, Moshi, Tanzania

Table S1. Real time PCR assays on the TaqMan Array Card used in MAL-ED.

All the assays have been described previously and extensively validated.[1–3] Nucleic acid was extracted with the QIAamp Fast DNA Stool mini kit (Qiagen, Hilden, Germany) with pre-treatment steps that included bead beating. AgPath One Step RT-PCR reagents were used for qPCR reactions, which were performed on ViiA 7 systems. Quantification cycles (Cqs) are the PCR cycle values at which fluorescence from amplification exceeds the background, which acts as an inverse metric of quantity of nucleic acid. Valid results required proper functioning of controls (the negative results of a sample are valid only when its external control MS2 is positive, Cq ≤ 35; the positive results are valid only when the corresponding extraction blank is negative for the relevant targets, Cq > 35), and excluded data flagged by the real time PCR software, i.e. BADROX in combination with NOISE or SPIKE.

	Pathogen	Gene
Viruses	Adenovirus 40/41	Fiber gene
	Astrovirus	Capsid
	Norovirus GI/GII	GI ORF1-2 and GII ORF1-2
	Rotavirus	*NSP3*
	Sapovirus	*RdRp*
Bacteria	EAEC*	*aaiC, aatA, aggR*
	Atypical EPEC*	*eae*
	Typical EPEC*	*bfpA*
	ETEC*	*LT, STh* and *STp*
	STEC*	*stx1, stx2*
	Aeromonas	Aerolysin
	Campylobacter spp.	*cadF* and *cpn60*
	Helicobacter pylori	*ureC*
	Plesiomonas shigelloides	*gyrB*
	Salmonella	*ttr*
	Shigella/EIEC†	*ipaH*
	Vibrio cholerae	*hlyA*
Fungi	*Enterocytozoon bieneusi*	*ITS*
	Encephalitozoon intestinalis	SSU rRNA
Protozoa	*Cryptosporidium*	18S rRNA
	Cyclospora cayetanensis	18S rRNA
	Cystoisospora belli	18S rRNA
	Entamoeba histolytica	18S rRNA
	Giardia	18S rRNA
Helminth	*Ancylostoma duodenale*	*ITS2*
	Ascaris lumbricoides	*ITS1*
	Necator americanus	*ITS2*
	Strongyloides stercoralis	Dispersed repetitive sequence
	Trichuris trichiura	18S rRNA
Controls	MS2	*MS2g1*
	PhHV	*gB*

**E. coli* pathotypes were defined as follows: EAEC (*aaiC*, or *aatA*, or both), atypical EPEC (*eae* without *bfpA*, *stx1*, and *stx2*), typical EPEC (*bfpA*), ETEC (STh, STp, or LT), STEC (*eae* without *bfpA* and with *stx1*, *stx2*, or both).
†Both *Shigella* and enteroinvasive *E. coli* are detected using the *ipaH* target, however based on previous findings that *S flexneri* and *S sonnei* account for the majority of ipaH detections,[2] plus the finding that *Shigella* culture positive stools are metagenomically similar to *ipaH* positive stools,[4] for simplicity we interpret *ipaH* detections as diagnostic of *Shigella*.

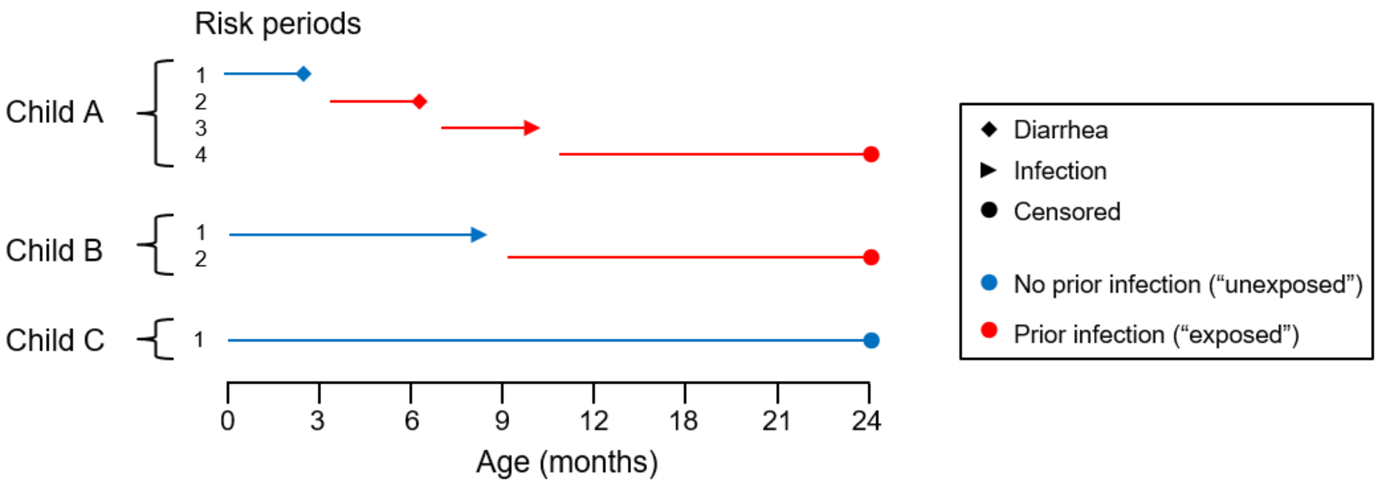

Figure S1. Conceptual diagram of the risk period formulation for example children A, B, and C in the Andersen and Gill extension of the Cox model. Each risk period is defined by birth or age at 21 days after a prior infection to age at subsequent outcome, and each child contributed multiple risk periods from birth to 2 years of age. This diagram was adapted from Kelly PJ and Lim LLY, 2000.[5]

Table S2. Estimates of protection against infection and attributable diarrhea due to one or two or more prior infections from the same pathogen in the MAL-ED cohort.

			Infection outcome				Attributable diarrhea outcome			
Pathogen	# previous infections	N	N (%) with subsequent infection	Unadjusted* HR (95% CI)	Adjusted† HR (95% CI)	Calibrated‡ HR (95% CI)	N (%) with subsequent diarrhea	Unadjusted* HR (95% CI)	Adjusted† HR (95% CI)	Calibrated‡ HR (95% CI)
Rotavirus	0	1715	839 (48.9)	1.	1.	1.	375 (21.9)	1.	1.	1.
	1	839	271 (32.3)	0.79 (0.68, 0.93)	0.79 (0.68, 0.93)	0.74 (0.61, 0.91)	127 (15.1)	0.62 (0.50, 0.78)	0.63 (0.50, 0.78)	0.53 (0.42, 0.66)
	2+	271	75 (27.7)	0.81 (0.61, 1.07)	0.81 (0.61, 1.07)	0.73 (0.55, 0.97)	42 (15.5)	0.56 (0.41, 0.78)	0.56 (0.40, 0.78)	0.43 (0.31, 0.61)
Astrovirus	0	1715	1048 (61.1)	1.	1.	1.	177 (10.3)	1.	1.	1.
	1	1048	482 (46.0)	0.83 (0.74, 0.93)	0.82 (0.73, 0.92)	0.78 (0.65, 0.94)	85 (8.1)	0.75 (0.57, 0.98)	0.75 (0.57, 0.98)	0.66 (0.49, 0.87)
	2+	482	173 (35.9)	0.75 (0.63, 0.88)	0.74 (0.62, 0.87)	0.72 (0.60, 0.88)	39 (8.1)	0.65 (0.44, 0.94)	0.64 (0.43, 0.94)	0.52 (0.35, 0.77)
Norovirus GII	0	1715	1310 (76.4)	1.	1.	1.	140 (8.2)	1.	1.	1.
	1	1310	799 (61.0)	0.98 (0.88, 1.08)	0.96 (0.87, 1.07)	0.96 (0.86, 1.07)	91 (6.9)	0.76 (0.57, 1.02)	0.77 (0.57, 1.02)	0.73 (0.52, 1.03)
	2+	799	386 (48.3)	0.87 (0.77, 0.98)	0.84 (0.74, 0.95)	0.88 (0.78, 1.01)	60 (7.5)	0.57 (0.41, 0.80)	0.57 (0.41, 0.80)	0.56 (0.40, 0.79)
Sapovirus	0	1715	1350 (78.7)	1.	1.	1.	229 (13.4)	1.	1.	1.
	1	1350	833 (61.7)	0.95 (0.87, 1.05)	0.93 (0.85, 1.03)	0.92 (0.82, 1.03)	158 (11.7)	0.94 (0.76, 1.16)	0.92 (0.75, 1.14)	0.82 (0.66, 1.02)
	2+	833	405 (48.6)	0.85 (0.76, 0.96)	0.83 (0.74, 0.94)	0.78 (0.69, 0.88)	120 (14.4)	1.06 (0.82, 1.35)	1.03 (0.80, 1.32)	0.84 (0.65, 1.09)
Adenovirus 40/41	0	1715	1161 (67.7)	1.	1.	1.	166 (9.7)	1.	1.	1.
	1	1161	591 (50.9)	0.97 (0.87, 1.09)	0.96 (0.86, 1.07)	0.89 (0.78, 1.01)	109 (9.4)	1.06 (0.82, 1.39)	1.03 (0.78, 1.34)	0.87 (0.65, 1.16)
	2+	591	286 (48.4)	1.04 (0.90, 1.22)	1.02 (0.87, 1.19)	0.95 (0.81, 1.13)	92 (15.6)	1.13 (0.83, 1.53)	1.07 (0.78, 1.46)	0.82 (0.59, 1.14)
Shigella	0	1715	1141 (66.5)	1.	1.	1.	391 (22.8)	1.	1.	1.
	1	1141	719 (63.0)	1.72 (1.54, 1.93)	1.69 (1.51, 1.90)	1.46 (1.21, 1.77)	171 (15.0)	1.09 (0.90, 1.33)	1.07 (0.87, 1.30)	0.85 (0.69, 1.04)
	2+	719	405 (56.3)	1.76 (1.57, 1.97)	1.70 (1.52, 1.91)	1.37 (0.95, 1.96)	126 (17.5)	1.00 (0.80, 1.24)	0.95 (0.76, 1.19)	0.67 (0.53, 0.84)
ST-ETEC	0	1715	1244 (72.5)	1.	1.	1.	137 (8.0)	1.	1.	1.
	1	1244	825 (66.3)	1.06 (0.96, 1.16)	1.05 (0.95, 1.16)	1.02 (0.92, 1.13)	144 (11.6)	1.45 (1.14, 1.85)	1.45 (1.13, 1.85)	1.25 (0.96, 1.63)
	2+	825	513 (62.2)	1.06 (0.95, 1.18)	1.04 (0.94, 1.16)	1.00 (0.90, 1.13)	116 (14.1)	1.13 (0.85, 1.51)	1.10 (0.82, 1.46)	0.84 (0.62, 1.13)
tEPEC	0	1715	1251 (72.9)	1.	1.	1.	23 (1.3)	1.	1.	1.
	1	1251	835 (66.7)	1.22 (1.10, 1.36)	1.21 (1.08, 1.34)	1.15 (0.94, 1.40)	6 (0.5)	1.01 (0.35, 2.95)	0.99 (0.35, 2.80)	0.60 (0.21, 1.75)
	2+	835	452 (54.1)	0.99 (0.88, 1.10)	0.96 (0.85, 1.07)	0.86 (0.66, 1.13)	2 (0.2)	0.59 (0.12, 2.96)	0.52 (0.11, 2.50)	0.46 (0.09, 2.48)
Campylobacter jejuni/coli	0	1715	1191 (69.4)	1.	1.	1.	73 (4.3)	1.	1.	1.
	1	1191	850 (71.4)	1.38 (1.24, 1.53)	1.35 (1.21, 1.50)	1.35 (1.20, 1.51)	37 (3.1)	1.30 (0.83, 2.04)	1.29 (0.82, 2.03)	1.34 (0.82, 2.16)
	2+	850	569 (66.9)	1.79 (1.61, 2.00)	1.71 (1.53, 1.91)	1.63 (1.45, 1.84)	40 (4.7)	1.54 (0.95, 2.49)	1.42 (0.87, 2.30)	1.43 (0.88, 2.34)
Cryptosporidium	0	1715	950 (55.4)	1.	1.	1.	86 (5.0)	1.	1.	1.
	1	950	347 (36.5)	0.93 (0.80, 1.07)	0.90 (0.78, 1.04)	0.81 (0.65, 1.02)	17 (1.8)	0.44 (0.26, 0.72)	0.42 (0.26, 0.70)	0.35 (0.21, 0.59)
	2+	347	111 (32.0)	0.88 (0.71, 1.07)	0.83 (0.68, 1.02)	0.71 (0.49, 1.03)	8 (2.3)	0.34 (0.16, 0.73)	0.32 (0.15, 0.70)	0.25 (0.11, 0.53)

*Hazard ratio adjusted for site

†Hazard ratio adjusted for site, SES, sex, enrollment WAZ, maternal education, maternal height, crowding, and exclusive breastfeeding in first 6 months; also shown in Figure 1A

‡Hazard ratio adjusted for the same variables above and calibrated based on negative control estimates; also shown in Figure 1B

Table S3. Estimates of protection against infection and attributable diarrhea due to one or more prior infections from the same pathogen in the MAL-ED cohort.

			Infection outcome				Attributable diarrhea outcome			
Pathogen	# previous infections	N	N (%) with subsequent infection	Unadjusted* HR (95% CI)	Adjusted† HR (95% CI)	Calibrated‡ HR (95% CI)	N (%) with subsequent diarrhea	Unadjusted* HR (95% CI)	Adjusted† HR (95% CI)	Calibrated‡ HR (95% CI)
Rotavirus	0	1715	839 (48.9)	1.	1.	1.	375 (21.9)	1.	1.	1.
	1+	839	271 (32.3)	0.80 (0.68, 0.93)	0.80 (0.68, 0.93)	0.74 (0.61, 0.89)	147 (17.5)	0.61 (0.50, 0.75)	0.61 (0.50, 0.75)	0.50 (0.41, 0.62)
Astrovirus	0	1715	1048 (61.1)	1.	1.	1.	177 (10.3)	1.	1.	1.
	1+	1048	482 (46.0)	0.81 (0.72, 0.90)	0.80 (0.71, 0.89)	0.77 (0.64, 0.92)	115 (11.0)	0.72 (0.56, 0.93)	0.72 (0.55, 0.93)	0.62 (0.48, 0.81)
Norovirus GII	0	1715	1310 (76.4)	1.	1.	1.	140 (8.2)	1.	1.	1.
	1+	1310	799 (61.0)	0.94 (0.86, 1.03)	0.92 (0.84, 1.01)	0.93 (0.84, 1.02)	138 (10.5)	0.69 (0.53, 0.91)	0.70 (0.53, 0.91)	0.67 (0.49, 0.91)
Sapovirus	0	1715	1350 (78.7)	1.	1.	1.	229 (13.4)	1.	1.	1.
	1+	1350	833 (61.7)	0.92 (0.84, 1.01)	0.90 (0.82, 0.98)	0.87 (0.79, 0.97)	237 (17.6)	0.98 (0.81, 1.19)	0.96 (0.79, 1.16)	0.84 (0.69, 1.03)
Adenovirus 40/41	0	1715	1161 (67.7)	1.	1.	1.	166 (9.7)	1.	1.	1.
	1+	1161	591 (50.9)	1.00 (0.89, 1.11)	0.98 (0.88, 1.09)	0.91 (0.80, 1.03)	154 (13.3)	1.09 (0.85, 1.40)	1.04 (0.81, 1.34)	0.86 (0.66, 1.13)
Shigella	0	1715	1141 (66.5)	1.	1.	1.	391 (22.8)	1.	1.	1.
	1+	1141	719 (63.0)	1.74 (1.58, 1.92)	1.70 (1.54, 1.87)	1.42 (1.07, 1.88)	250 (21.9)	1.05 (0.89, 1.25)	1.02 (0.85, 1.21)	0.79 (0.65, 0.95)
ST-ETEC	0	1715	1244 (72.5)	1.	1.	1.	137 (8.0)	1.	1.	1.
	1+	1244	825 (66.3)	1.06 (0.97, 1.16)	1.05 (0.96, 1.15)	1.02 (0.93, 1.13)	234 (18.8)	1.33 (1.06, 1.68)	1.31 (1.04, 1.66)	1.11 (0.85, 1.44)
tEPEC	0	1715	1251 (72.9)	1.	1.	1.	23 (1.3)	1.	1.	1.
	1+	1251	835 (66.7)	1.13 (1.03, 1.23)	1.10 (1.01, 1.21)	1.06 (0.88, 1.27)	8 (0.6)	0.88 (0.31, 2.52)	0.84 (0.31, 2.29)	0.60 (0.22, 1.65)
Campylobacter jejuni/coli	0	1715	1191 (69.4)	1.	1.	1.	73 (4.3)	1.	1.	1.
	1+	1191	850 (71.4)	1.56 (1.42, 1.71)	1.50 (1.36, 1.65)	1.47 (1.33, 1.64)	68 (5.7)	1.39 (0.93, 2.09)	1.34 (0.89, 2.02)	1.42 (0.93, 2.15)
Cryptosporidium	0	1715	950 (55.4)	1.	1.	1.	86 (5.0)	1.	1.	1.
	1+	950	347 (36.5)	0.91 (0.80, 1.05)	0.88 (0.77, 1.01)	0.79 (0.61, 1.04)	24 (2.5)	0.40 (0.25, 0.64)	0.39 (0.24, 0.62)	0.32 (0.20, 0.51)

*Hazard ratio adjusted for site
†Hazard ratio adjusted for site, SES, sex, enrollment WAZ, maternal education, maternal height, crowding, and exclusive breastfeeding in first 6 months
‡Hazard ratio adjusted for the same variables above and calibrated based on negative control estimates

Table S4. Site-specific estimates of protection against infection and attributable diarrhea due to one or more prior infections from the same pathogen in the MAL-ED cohort.

		Infection outcome				Attributable diarrhea outcome			
Pathogen	Site	N total infections	Unadjusted* HR (95% CI)	Adjusted† HR (95% CI)	Calibrated‡ HR (95% CI)	N total diarrhea episodes	Unadjusted* HR (95% CI)	Adjusted† HR (95% CI)	Calibrated‡ HR (95% CI)
Rotavirus	Dhaka, Bangladesh	347	0.66 (0.53, 0.83)	0.67 (0.53, 0.83)	0.62 (0.48, 0.79)	216	0.62 (0.46, 0.83)	0.62 (0.47, 0.83)	0.52 (0.38, 0.69)
	Fortaleza, Brazil	25	0.99 (0.14, 6.96)	0.99 (0.14, 6.93)	0.92 (0.13, 6.45)	3	—§	—§	—§
	Vellore, India	187	1.05 (0.76, 1.46)	1.04 (0.74, 1.45)	0.96 (0.68, 1.37)	67	0.83 (0.52, 1.34)	0.83 (0.51, 1.34)	0.68 (0.42, 1.11)
	Bhaktapur, Nepal	180	0.84 (0.61, 1.16)	0.84 (0.61, 1.16)	0.78 (0.55, 1.10)	97	0.53 (0.32, 0.86)	0.52 (0.32, 0.85)	0.43 (0.26, 0.71)
	Loreto, Peru	130	0.75 (0.49, 1.17)	0.76 (0.49, 1.18)	0.71 (0.45, 1.11)	63	0.80 (0.43, 1.50)	0.81 (0.43, 1.52)	0.67 (0.36, 1.26)
	N.F., Pakistan	165	0.51 (0.31, 0.82)	0.50 (0.31, 0.81)	0.46 (0.28, 0.76)	81	0.38 (0.20, 0.71)	0.38 (0.20, 0.71)	0.31 (0.17, 0.59)
	Venda, South Africa	46	0.46 (0.12, 1.81)	0.46 (0.11, 1.81)	0.42 (0.11, 1.69)	6	—	—	—
	Haydom, Tanzania	137	1.50 (0.96, 2.34)	1.48 (0.95, 2.31)	1.38 (0.87, 2.18)	19	0.49 (0.14, 1.71)	0.49 (0.14, 1.70)	0.40 (0.12, 1.41)
Astrovirus	Dhaka, Bangladesh	374	0.74 (0.60, 0.91)	0.73 (0.59, 0.91)	0.71 (0.55, 0.91)	45	0.76 (0.42, 1.37)	0.75 (0.42, 1.35)	0.65 (0.36, 1.18)
	Fortaleza, Brazil	23	0.00 (0.00, 0.00)	0.00 (0.00, 0.00)	0.00 (0.00, 0.00)	3	—	—	—
	Vellore, India	215	1.09 (0.79, 1.49)	1.05 (0.77, 1.45)	1.02 (0.72, 1.44)	37	0.54 (0.25, 1.15)	0.54 (0.25, 1.18)	0.47 (0.22, 1.03)
	Bhaktapur, Nepal	180	0.89 (0.64, 1.22)	0.89 (0.65, 1.22)	0.86 (0.61, 1.21)	39	0.99 (0.51, 1.92)	1.01 (0.52, 1.98)	0.88 (0.45, 1.73)
	Loreto, Peru	352	0.88 (0.73, 1.08)	0.87 (0.72, 1.06)	0.84 (0.66, 1.07)	110	0.94 (0.64, 1.37)	0.92 (0.63, 1.35)	0.80 (0.55, 1.18)
	N.F., Pakistan	363	0.71 (0.57, 0.88)	0.70 (0.56, 0.87)	0.67 (0.52, 0.88)	57	0.48 (0.28, 0.83)	0.47 (0.27, 0.83)	0.41 (0.23, 0.72)
	Venda, South Africa	110	1.10 (0.63, 1.90)	1.09 (0.63, 1.87)	1.05 (0.60, 1.83)	4	—	—	—
	Haydom, Tanzania	142	0.57 (0.39, 0.81)	0.56 (0.39, 0.81)	0.54 (0.37, 0.80)	7	—	—	—
Norovirus GII	Dhaka, Bangladesh	404	1.04 (0.85, 1.28)	1.01 (0.83, 1.24)	1.02 (0.83, 1.25)	43	1.31 (0.67, 2.57)	1.26 (0.64, 2.48)	1.21 (0.61, 2.41)
	Fortaleza, Brazil	61	0.81 (0.38, 1.74)	0.78 (0.37, 1.68)	0.79 (0.37, 1.70)	11	0.99 (0.21, 4.66)	0.97 (0.20, 4.59)	0.93 (0.20, 4.43)
	Vellore, India	346	0.93 (0.75, 1.14)	0.92 (0.75, 1.13)	0.92 (0.75, 1.14)	13	0.90 (0.30, 2.72)	0.90 (0.30, 2.73)	0.86 (0.28, 2.65)
	Bhaktapur, Nepal	321	1.15 (0.92, 1.43)	1.14 (0.91, 1.42)	1.15 (0.92, 1.44)	68	0.69 (0.41, 1.15)	0.71 (0.42, 1.18)	0.68 (0.40, 1.15)
	Loreto, Peru	492	1.07 (0.92, 1.25)	1.05 (0.90, 1.23)	1.06 (0.90, 1.24)	119	0.64 (0.44, 0.94)	0.66 (0.45, 0.97)	0.63 (0.42, 0.95)
	N.F., Pakistan	639	0.90 (0.76, 1.07)	0.89 (0.74, 1.06)	0.89 (0.75, 1.07)	13	0.38 (0.13, 1.15)	0.39 (0.13, 1.16)	0.37 (0.12, 1.13)
	Venda, South Africa	169	0.78 (0.55, 1.11)	0.77 (0.54, 1.10)	0.78 (0.54, 1.11)	9	—	—	—
	Haydom, Tanzania	285	0.67 (0.53, 0.86)	0.66 (0.51, 0.84)	0.66 (0.52, 0.85)	17	0.48 (0.18, 1.28)	0.47 (0.18, 1.24)	0.45 (0.17, 1.20)
Sapovirus	Dhaka, Bangladesh	512	1.07 (0.90, 1.28)	1.05 (0.88, 1.25)	1.02 (0.85, 1.22)	91	1.45 (0.91, 2.30)	1.40 (0.88, 2.23)	1.23 (0.77, 1.96)
	Fortaleza, Brazil	69	1.58 (0.91, 2.76)	1.56 (0.89, 2.74)	1.52 (0.87, 2.66)	8	—	—	—
	Vellore, India	361	0.70 (0.56, 0.87)	0.68 (0.55, 0.85)	0.66 (0.53, 0.83)	64	0.56 (0.33, 0.94)	0.54 (0.32, 0.92)	0.48 (0.28, 0.81)
	Bhaktapur, Nepal	350	1.08 (0.89, 1.32)	1.06 (0.87, 1.30)	1.03 (0.84, 1.27)	95	1.07 (0.73, 1.56)	1.05 (0.72, 1.54)	0.93 (0.63, 1.36)
	Loreto, Peru	445	1.08 (0.90, 1.30)	1.05 (0.87, 1.25)	1.02 (0.84, 1.23)	187	1.09 (0.82, 1.47)	1.07 (0.80, 1.44)	0.94 (0.70, 1.27)
	N.F., Pakistan	544	0.73 (0.61, 0.88)	0.71 (0.59, 0.85)	0.69 (0.57, 0.84)	58	0.68 (0.39, 1.18)	0.65 (0.37, 1.13)	0.57 (0.33, 1.00)
	Venda, South Africa	237	1.09 (0.83, 1.43)	1.07 (0.82, 1.41)	1.04 (0.79, 1.37)	8	—	—	—
	Haydom, Tanzania	283	0.81 (0.63, 1.03)	0.80 (0.63, 1.02)	0.78 (0.60, 1.00)	15	0.67 (0.25, 1.78)	0.66 (0.25, 1.76)	0.58 (0.22, 1.55)

Adenovirus 40/41	Dhaka, Bangladesh	548	0.96 (0.79, 1.17)	0.95 (0.78, 1.14)	0.88 (0.72, 1.07)	283	1.14 (0.86, 1.51)	1.09 (0.82, 1.44)	0.90 (0.67, 1.22)
	Fortaleza, Brazil	69	0.54 (0.28, 1.04)	0.53 (0.27, 1.03)	0.49 (0.25, 0.96)	5	—	—	—
	Vellore, India	330	1.06 (0.81, 1.39)	1.04 (0.80, 1.36)	0.97 (0.74, 1.27)	26	0.80 (0.35, 1.84)	0.76 (0.33, 1.76)	0.63 (0.27, 1.47)
	Bhaktapur, Nepal	167	0.92 (0.63, 1.33)	0.92 (0.63, 1.33)	0.85 (0.58, 1.24)	12	0.00 (0.00, 0.00)	0.00 (0.00, 0.00)	0.00 (0.00, 0.00)
	Loreto, Peru	498	1.10 (0.91, 1.33)	1.08 (0.90, 1.30)	1.01 (0.83, 1.22)	51	1.25 (0.73, 2.13)	1.21 (0.70, 2.07)	1.00 (0.58, 1.73)
	N.F., Pakistan	318	1.04 (0.83, 1.29)	1.01 (0.80, 1.27)	0.94 (0.74, 1.19)	0	—	—	—
	Venda, South Africa	131	1.00 (0.65, 1.54)	1.00 (0.65, 1.53)	0.93 (0.60, 1.43)	4	—	—	—
	Haydom, Tanzania	203	0.85 (0.60, 1.21)	0.84 (0.59, 1.20)	0.78 (0.55, 1.12)	9	—	—	—
Shigella	Dhaka, Bangladesh	556	1.57 (1.31, 1.89)	1.52 (1.27, 1.83)	1.28 (0.93, 1.76)	259	0.86 (0.67, 1.09)	0.83 (0.65, 1.06)	0.64 (0.50, 0.82)
	Fortaleza, Brazil	72	2.29 (1.25, 4.21)	2.18 (1.19, 3.98)	1.82 (0.94, 3.52)	12	1.76 (0.46, 6.71)	1.74 (0.46, 6.56)	1.34 (0.36, 5.07)
	Vellore, India	410	1.31 (1.06, 1.63)	1.26 (1.02, 1.56)	1.06 (0.75, 1.48)	95	0.89 (0.59, 1.33)	0.88 (0.58, 1.32)	0.68 (0.45, 1.02)
	Bhaktapur, Nepal	242	3.30 (2.51, 4.33)	3.28 (2.49, 4.32)	2.75 (1.88, 4.02)	78	1.15 (0.72, 1.86)	1.12 (0.70, 1.80)	0.86 (0.54, 1.40)
	Loreto, Peru	487	3.02 (2.50, 3.65)	2.94 (2.43, 3.56)	2.46 (1.78, 3.40)	150	1.90 (1.34, 2.69)	1.83 (1.29, 2.60)	1.41 (0.99, 2.02)
	N.F., Pakistan	190	1.31 (0.99, 1.74)	1.31 (0.98, 1.74)	1.09 (0.74, 1.61)	102	0.99 (0.65, 1.53)	0.94 (0.60, 1.45)	0.72 (0.47, 1.12)
	Venda, South Africa	152	1.43 (1.03, 1.99)	1.42 (1.02, 1.97)	1.19 (0.78, 1.81)	10	0.73 (0.15, 3.46)	0.73 (0.15, 3.46)	0.57 (0.12, 2.67)
	Haydom, Tanzania	539	1.16 (0.95, 1.42)	1.14 (0.94, 1.39)	0.96 (0.69, 1.33)	13	0.16 (0.05, 0.55)	0.16 (0.05, 0.55)	0.13 (0.04, 0.42)
ST-ETEC	Dhaka, Bangladesh	905	1.02 (0.87, 1.20)	1.01 (0.86, 1.19)	0.99 (0.83, 1.17)	205	0.98 (0.68, 1.41)	0.96 (0.66, 1.39)	0.81 (0.55, 1.20)
	Fortaleza, Brazil	43	1.50 (0.71, 3.14)	1.45 (0.69, 3.05)	1.42 (0.67, 2.97)	4	—	—	—
	Vellore, India	379	0.86 (0.70, 1.05)	0.85 (0.70, 1.04)	0.83 (0.67, 1.02)	55	1.00 (0.59, 1.69)	0.98 (0.58, 1.66)	0.83 (0.48, 1.42)
	Bhaktapur, Nepal	463	1.09 (0.91, 1.31)	1.09 (0.91, 1.31)	1.06 (0.88, 1.28)	66	2.28 (1.32, 3.93)	2.29 (1.32, 3.98)	1.93 (1.10, 3.40)
	Loreto, Peru	340	1.30 (1.06, 1.59)	1.27 (1.04, 1.56)	1.24 (1.01, 1.52)	56	1.77 (1.06, 2.96)	1.75 (1.04, 2.94)	1.47 (0.86, 2.52)
	N.F., Pakistan	292	1.17 (0.92, 1.49)	1.15 (0.90, 1.46)	1.12 (0.88, 1.43)	31	1.59 (0.80, 3.18)	1.56 (0.78, 3.12)	1.31 (0.65, 2.66)
	Venda, South Africa	107	1.00 (0.61, 1.62)	0.98 (0.60, 1.60)	0.96 (0.59, 1.56)	4	—	—	—
	Haydom, Tanzania	687	1.02 (0.85, 1.22)	1.00 (0.84, 1.21)	0.98 (0.81, 1.18)	25	0.81 (0.36, 1.80)	0.80 (0.36, 1.79)	0.68 (0.30, 1.53)
tEPEC	Dhaka, Bangladesh	617	1.15 (0.98, 1.35)	1.13 (0.96, 1.32)	1.08 (0.86, 1.35)	2	—	—	—
	Fortaleza, Brazil	50	1.34 (0.65, 2.74)	1.31 (0.64, 2.71)	1.26 (0.60, 2.63)	0	—	—	—
	Vellore, India	510	0.97 (0.81, 1.17)	0.96 (0.79, 1.16)	0.91 (0.71, 1.17)	8	—	—	—
	Bhaktapur, Nepal	304	1.80 (1.44, 2.23)	1.77 (1.42, 2.19)	1.69 (1.29, 2.21)	5	—	—	—
	Loreto, Peru	436	1.77 (1.47, 2.13)	1.72 (1.43, 2.07)	1.64 (1.29, 2.10)	2	—	—	—
	N.F., Pakistan	307	0.81 (0.64, 1.01)	0.79 (0.63, 1.00)	0.76 (0.57, 1.00)	11	0.55 (0.11, 2.75)	0.52 (0.11, 2.49)	0.37 (0.08, 1.78)
	Venda, South Africa	110	1.03 (0.66, 1.58)	1.02 (0.66, 1.57)	0.97 (0.61, 1.55)	1	—	—	—
	Haydom, Tanzania	515	0.79 (0.64, 0.98)	0.79 (0.64, 0.97)	0.75 (0.58, 0.98)	2	—	—	—
Campylobacter jejuni/coli	Dhaka, Bangladesh	804	1.77 (1.49, 2.11)	1.70 (1.42, 2.02)	1.67 (1.39, 2.00)	1	—	—	—
	Fortaleza, Brazil	32	2.10 (0.88, 5.03)	2.01 (0.83, 4.84)	1.97 (0.82, 4.77)	5	—	—	—
	Vellore, India	281	1.36 (1.03, 1.82)	1.30 (0.98, 1.73)	1.28 (0.96, 1.71)	28	1.97 (0.90, 4.31)	1.86 (0.86, 4.06)	1.97 (0.90, 4.31)
	Bhaktapur, Nepal	677	1.54 (1.30, 1.83)	1.50 (1.26, 1.79)	1.48 (1.24, 1.77)	2	—	—	—
	Loreto, Peru	552	1.34 (1.12, 1.59)	1.30 (1.09, 1.54)	1.28 (1.07, 1.53)	109	1.14 (0.72, 1.81)	1.10 (0.70, 1.75)	1.16 (0.73, 1.86)
	N.F., Pakistan	266	1.40 (1.05, 1.85)	1.32 (0.99, 1.75)	1.30 (0.97, 1.73)	4	—	—	—
	Venda, South Africa	84	1.69 (1.06, 2.69)	1.68 (1.06, 2.67)	1.65 (1.04, 2.63)	6	—	—	—

	Haydom, Tanzania	900	1.70 (1.38, 2.10)	1.64 (1.33, 2.02)	1.61 (1.30, 1.99)	1	0.00 (0.00, 0.00)	0.00 (0.00, 0.00)	0.00 (0.00, 0.00)
Cryptosporidium	Dhaka, Bangladesh	212	1.08 (0.79, 1.46)	1.05 (0.77, 1.43)	0.95 (0.65, 1.39)	18	0.33 (0.09, 1.14)	0.31 (0.09, 1.07)	0.25 (0.07, 0.88)
	Fortaleza, Brazil	14	0.00 (0.00, 0.00)	0.00 (0.00, 0.00)	0.00 (0.00, 0.00)	0	—	—	—
	Vellore, India	141	0.64 (0.44, 0.94)	0.61 (0.42, 0.90)	0.55 (0.35, 0.86)	15	0.33 (0.08, 1.37)	0.31 (0.08, 1.28)	0.25 (0.06, 1.05)
	Bhaktapur, Nepal	174	1.72 (1.21, 2.44)	1.67 (1.17, 2.37)	1.51 (0.99, 2.29)	13	0.29 (0.04, 2.30)	0.28 (0.03, 2.24)	0.22 (0.03, 1.83)
	Loreto, Peru	342	1.27 (1.02, 1.58)	1.21 (0.97, 1.51)	1.09 (0.80, 1.50)	49	0.47 (0.26, 0.84)	0.46 (0.25, 0.84)	0.37 (0.20, 0.68)
	N.F., Pakistan	180	0.57 (0.40, 0.82)	0.54 (0.37, 0.78)	0.49 (0.32, 0.75)	14	0.50 (0.15, 1.66)	0.50 (0.15, 1.63)	0.40 (0.12, 1.33)
	Venda, South Africa	91	0.39 (0.17, 0.89)	0.38 (0.17, 0.88)	0.35 (0.15, 0.82)	0	—	—	—
	Haydom, Tanzania	299	0.70 (0.56, 0.89)	0.69 (0.54, 0.87)	0.62 (0.45, 0.86)	2	—	—	—

*Hazard ratio adjusted for site

†Hazard ratio adjusted for site, SES, sex, enrollment WAZ, maternal education, maternal height, crowding, and exclusive breastfeeding in first 6 months

‡Hazard ratio adjusted for the same variables above and calibrated based on negative control estimates calculated across sites

§Not estimated if fewer than 10 total diarrhea episodes at the site

N. F., Pakistan = Naushero Feroze, Pakistan

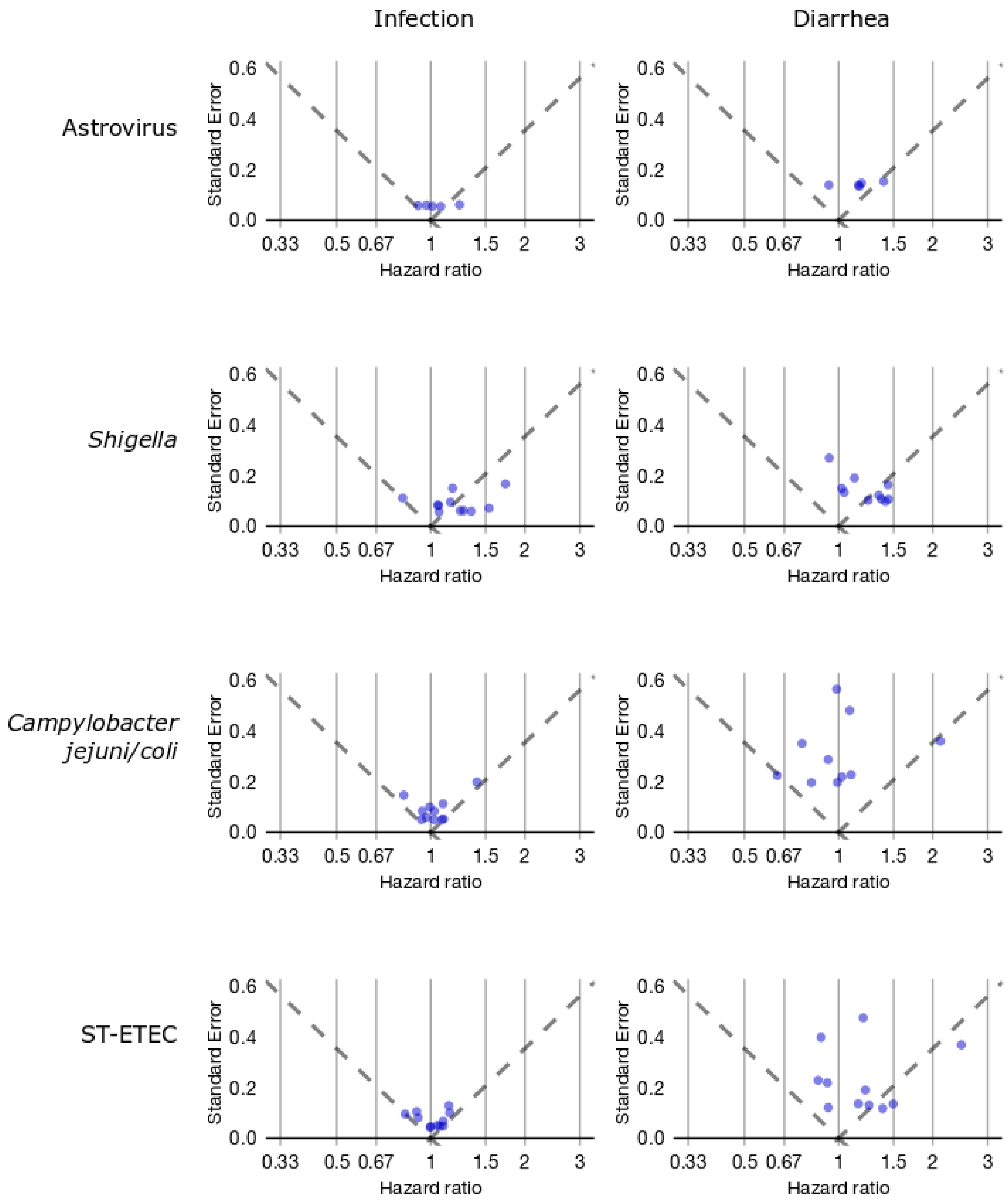

Figure S2. Funnel plots of the negative control estimates. Each dot represents one negative control association between 1+ prior infections with another pathogen of the same type (bacteria, viruses, parasites; i.e. a negative control pathogen) and subsequent infection or diarrhea due to the pathogen of interest (listed on left). Extreme estimates were excluded (hazard ratio < 0.001 or standard error ≥ 0.8). For the expected null distribution, the negative control estimates are expected to be centered around a hazard ratio of 1 and 95% of the estimates are expected to lie between the two dotted gray lines. Deviations from this expected distribution indicate the direction and magnitude of systematic error.

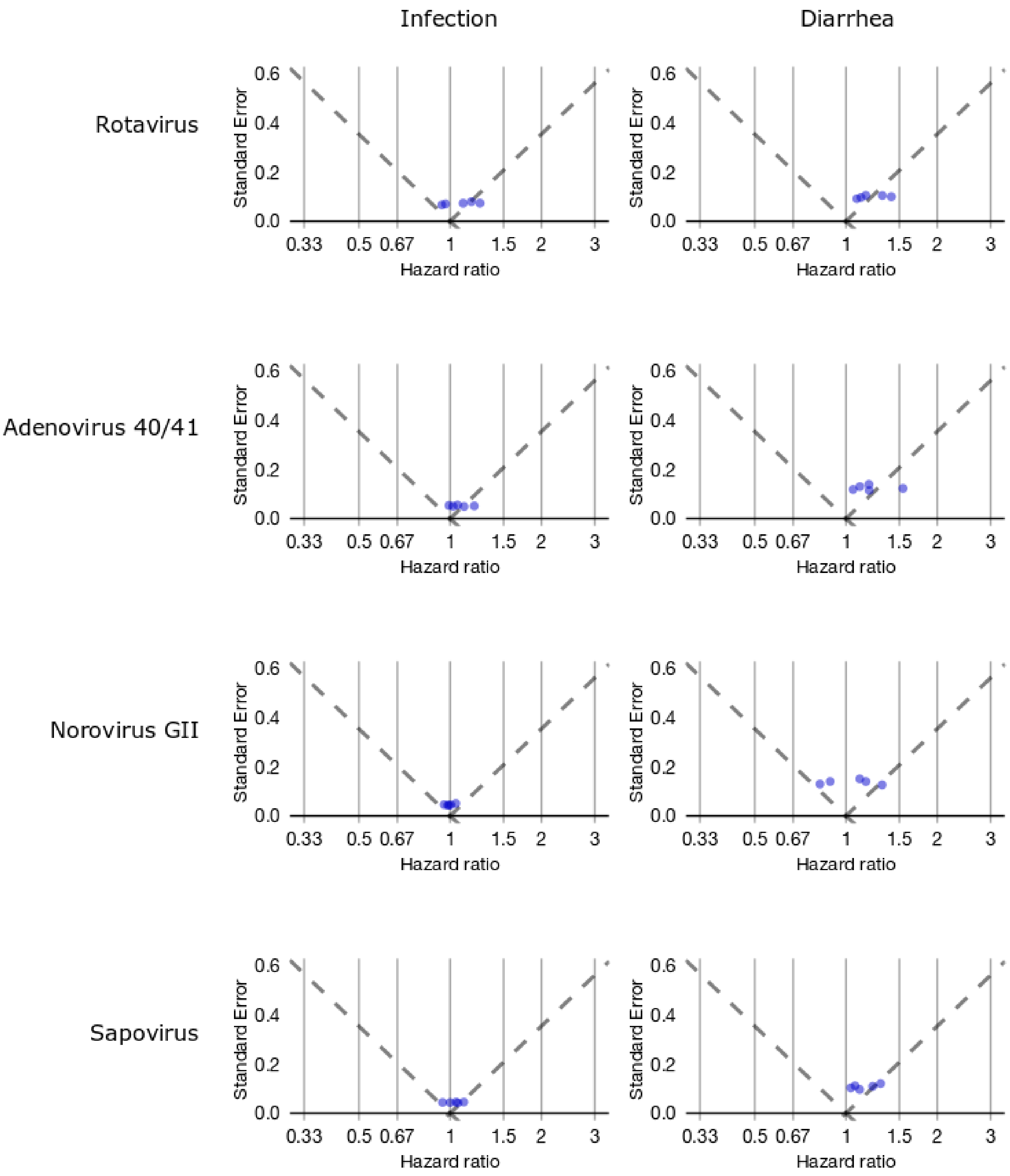

Figure S2 continued.

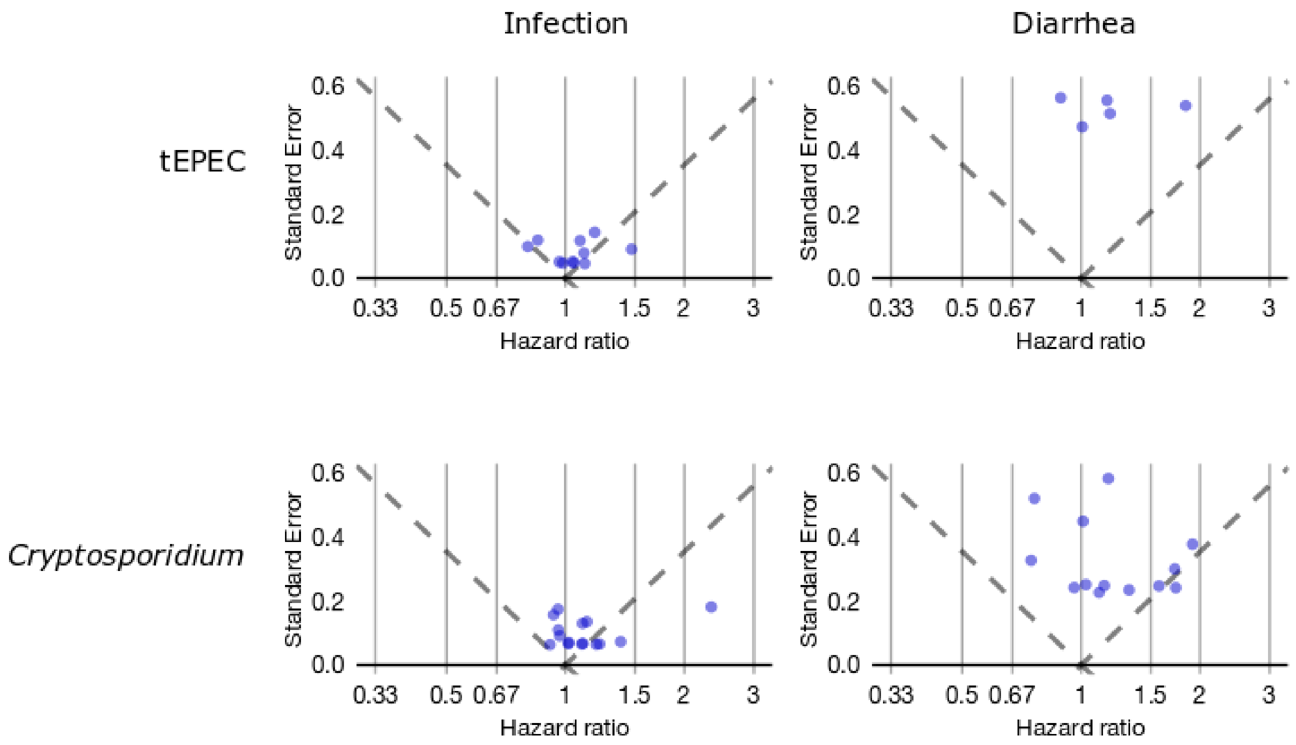

Figure S2 continued.

Sensitivity analyses

Table S5. Age-specific estimates of protection against infection and attributable diarrhea due to one or more prior infections from the same pathogen in the MAL-ED cohort.

			Infection outcome				Attributable diarrhea outcome		
Pathogen	Age (months)	# previous infections*	N	N (%) with subsequent infection	HR† (95% CI)	Calibrated‡ HR (95% CI)	N (%) with subsequent diarrhea	HR† (95% CI)	Calibrated‡ HR (95% CI)
Rotavirus	0-11	0	1715	575 (33.5)	1.	1.	246 (14.3)	1.	1.
		1+	563	123 (21.8)	0.80 (0.65, 0.98)	0.73 (0.59, 0.91)	68 (12.1)	0.64 (0.49, 0.84)	0.53 (0.40, 0.70)
	12-23	0	1140	264 (23.2)	1.	1.	129 (11.3)	1.	1.
		1+	839	200 (23.8)	0.83 (0.67, 1.02)	0.75 (0.58, 0.97)	98 (11.7)	0.62 (0.46, 0.82)	0.50 (0.37, 0.68)
Astrovirus	0-11	0	1715	662 (38.6)	1.	1.	104 (6.1)	1.	1.
		1+	645	149 (23.1)	0.75 (0.62, 0.89)	0.72 (0.58, 0.89)	28 (4.4)	0.74 (0.48, 1.15)	0.60 (0.38, 0.94)
	12-23	0	1053	386 (36.7)	1.	1.	73 (6.9)	1.	1.
		1+	1047	398 (38.0)	0.83 (0.71, 0.96)	0.80 (0.68, 0.93)	92 (8.8)	0.68 (0.49, 0.94)	0.62 (0.44, 0.87)
Norovirus GII	0-11	0	1715	1038 (60.5)	1.	1.	99 (5.8)	1.	1.
		1+	1022	437 (42.8)	0.97 (0.86, 1.09)	0.93 (0.83, 1.05)	51 (5.0)	0.72 (0.50, 1.04)	0.61 (0.40, 0.93)
	12-23	0	677	272 (40.2)	1.	1.	41 (6.1)	1.	1.
		1+	1310	589 (45.0)	0.82 (0.71, 0.96)	0.86 (0.74, 1.00)	92 (7.0)	0.58 (0.38, 0.89)	0.64 (0.41, 0.99)
Sapovirus	0-11	0	1715	837 (48.8)	1.	1.	145 (8.5)	1.	1.
		1+	804	255 (31.7)	0.93 (0.80, 1.07)	0.87 (0.75, 1.01)	47 (5.9)	0.68 (0.48, 0.95)	0.55 (0.39, 0.78)
	12-23	0	878	513 (58.4)	1.	1.	84 (9.6)	1.	1.
		1+	1349	748 (55.4)	0.87 (0.77, 0.97)	0.87 (0.76, 1.00)	212 (15.7)	1.13 (0.86, 1.48)	1.07 (0.81, 1.42)
Adenovirus 40/41	0-11	0	1715	720 (42.0)	1.	1.	129 (7.5)	1.	1.
		1+	705	230 (32.6)	1.15 (0.98, 1.35)	1.11 (0.94, 1.31)	69 (9.8)	1.09 (0.79, 1.50)	0.95 (0.68, 1.31)
	12-23	0	995	441 (44.3)	1.	1.	37 (3.7)	1.	1.
		1+	1160	500 (43.1)	0.85 (0.74, 0.98)	0.78 (0.66, 0.93)	108 (9.3)	0.96 (0.65, 1.41)	0.76 (0.51, 1.14)
Shigella	0-11	0	1715	401 (23.4)	1.	1.	117 (6.8)	1.	1.
		1+	388	123 (31.7)	2.59 (2.05, 3.26)	2.01 (1.57, 2.57)	21 (5.4)	1.39 (0.85, 2.29)	0.85 (0.52, 1.40)
	12-23	0	1314	740 (56.3)	1.	1.	274 (20.9)	1.	1.
		1+	1134	689 (60.8)	1.53 (1.37, 1.69)	1.35 (1.10, 1.66)	234 (20.7)	0.97 (0.80, 1.17)	0.75 (0.61, 0.91)
ST-ETEC	0-11	0	1715	768 (44.8)	1.	1.	92 (5.4)	1.	1.
		1+	758	285 (37.6)	1.08 (0.94, 1.24)	1.05 (0.91, 1.21)	60 (7.9)	0.96 (0.69, 1.35)	0.81 (0.57, 1.15)
	12-23	0	947	476 (50.3)	1.	1.	45 (4.8)	1.	1.
		1+	1244	775 (62.3)	1.01 (0.90, 1.14)	1.00 (0.88, 1.13)	190 (15.3)	1.63 (1.16, 2.31)	1.38 (0.95, 1.99)
tEPEC	0-11	0	1715	913 (53.2)	1.	1.	20 (1.2)	1.	1.
		1+	899	393 (43.7)	1.15 (1.01, 1.31)	1.16 (0.95, 1.42)	6 (0.7)	1.23 (0.46, 3.29)	0.77 (0.28, 2.16)
	12-23	0	802	338 (42.1)	1.	1.	3 (0.4)	1.	1.

		1+	1250	680 (54.4)	0.97 (0.85, 1.12)	0.92 (0.80, 1.06)	2 (0.2)	0.23 (0.06, 0.92)	0.16 (0.04, 0.66)
Campylobacter jejuni/coli	0-11	0	1715	927 (54.1)	1.	1.	61 (3.6)	1.	1.
		1+	909	476 (52.4)	1.62 (1.43, 1.83)	1.60 (1.41, 1.82)	33 (3.6)	1.36 (0.83, 2.22)	1.33 (0.79, 2.22)
	12-23	0	788	264 (33.5)	1.	1.	12 (1.5)	1.	1.
		1+	1191	702 (58.9)	1.26 (1.08, 1.47)	1.26 (1.06, 1.49)	43 (3.6)	1.08 (0.51, 2.30)	1.14 (0.53, 2.45)
Cryptosporidium	0-11	0	1715	339 (19.8)	1.	1.	32 (1.9)	1.	1.
		1+	324	63 (19.4)	1.76 (1.28, 2.42)	1.52 (0.95, 2.43)	32 (9.9)	0.00 (0.00, 0.00)	0.00 (0.00, 0.00)
	12-23	0	1376	611 (44.4)	1.	1.	54 (3.9)	1.	1.
		1+	948	312 (32.9)	0.75 (0.66, 0.87)	0.71 (0.61, 0.82)	24 (2.5)	0.43 (0.26, 0.71)	0.35 (0.21, 0.58)

*Previous infections from age 0-11 months were included in the exposure classification for the 12-23 month age group
†Hazard ratio adjusted for site, SES, sex, enrollment WAZ, maternal education, maternal height, crowding, and exclusive breastfeeding in first 6 months
‡Hazard ratio adjusted for the same variables above and calibrated based on negative control estimates

Table S6. Estimates of protection with cut-off for pathogen quantity detected to define infections at Cq < 35 instead of Cq ≤ 30.

			Infection outcome			Attributable diarrhea outcome		
Pathogen	# previous infections	N	N (%) with subsequent infection	HR* (95% CI)	Calibrated† HR (95% CI)	N (%) with subsequent diarrhea	HR* (95% CI)	Calibrated† HR (95% CI)
Rotavirus	0	1715	1098 (64.0)	1.	1.	294 (17.1)	1.	1.
	1	1098	530 (48.3)	0.99 (0.87, 1.11)	0.92 (0.77, 1.10)	139 (12.7)	0.71 (0.57, 0.89)	0.60 (0.48, 0.76)
	2+	530	222 (41.9)	1.07 (0.91, 1.26)	0.97 (0.81, 1.16)	92 (17.4)	0.72 (0.56, 0.93)	0.56 (0.42, 0.73)
Astrovirus	0	1715	1376 (80.2)	1.	1.	82 (4.8)	1.	1.
	1	1376	1023 (74.3)	1.15 (1.05, 1.26)	1.09 (0.92, 1.30)	80 (5.8)	1.19 (0.85, 1.66)	1.04 (0.74, 1.46)
	2+	1023	773 (75.6)	1.24 (1.11, 1.38)	1.22 (1.05, 1.40)	120 (11.7)	0.93 (0.65, 1.35)	0.76 (0.52, 1.10)
Norovirus GII	0	1715	1561 (91.0)	1.	1.	120 (7.0)	1.	1.
	1	1561	1298 (83.2)	1.26 (1.15, 1.37)	1.25 (1.14, 1.37)	65 (4.2)	0.58 (0.41, 0.81)	0.55 (0.38, 0.81)
	2+	1298	953 (73.4)	1.04 (0.95, 1.14)	1.10 (1.00, 1.22)	98 (7.6)	0.42 (0.29, 0.59)	0.41 (0.29, 0.58)
Sapovirus	0	1715	1579 (92.1)	1.	1.	150 (8.7)	1.	1.
	1	1579	1329 (84.2)	1.15 (1.06, 1.25)	1.14 (1.03, 1.25)	126 (8.0)	0.89 (0.70, 1.13)	0.79 (0.62, 1.01)
	2+	1329	1003 (75.5)	1.09 (1.00, 1.19)	1.02 (0.93, 1.12)	203 (15.3)	0.77 (0.59, 1.01)	0.63 (0.48, 0.83)
Adenovirus 40/41	0	1715	1508 (87.9)	1.	1.	88 (5.1)	1.	1.
	1	1508	1158 (76.8)	1.13 (1.04, 1.23)	1.04 (0.94, 1.16)	70 (4.6)	0.88 (0.62, 1.25)	0.75 (0.52, 1.07)
	2+	1158	858 (74.1)	1.35 (1.23, 1.49)	1.27 (1.13, 1.42)	163 (14.1)	1.03 (0.72, 1.47)	0.79 (0.55, 1.14)
Shigella	0	1715	1407 (82.0)	1.	1.	311 (18.1)	1.	1.
	1	1407	1047 (74.4)	1.60 (1.46, 1.76)	1.38 (1.16, 1.66)	164 (11.7)	0.92 (0.75, 1.12)	0.73 (0.59, 0.90)
	2+	1047	723 (69.1)	1.99 (1.81, 2.19)	1.60 (1.12, 2.28)	192 (18.3)	0.93 (0.75, 1.14)	0.65 (0.52, 0.81)
ST-ETEC	0	1715	1453 (84.7)	1.	1.	71 (4.1)	1.	1.
	1	1453	1172 (80.7)	1.14 (1.05, 1.24)	1.11 (1.01, 1.22)	97 (6.7)	1.62 (1.17, 2.24)	1.39 (0.99, 1.96)
	2+	1172	912 (77.8)	1.19 (1.09, 1.30)	1.14 (1.04, 1.26)	204 (17.4)	1.65 (1.19, 2.28)	1.26 (0.90, 1.76)
tEPEC	0	1715	1458 (85.0)	1.	1.	19 (1.1)	1.	1.
	1	1458	1159 (79.5)	1.23 (1.12, 1.35)	1.18 (0.97, 1.43)	7 (0.5)	1.15 (0.45, 2.93)	0.70 (0.27, 1.84)
	2+	1159	846 (73.0)	1.15 (1.04, 1.26)	1.03 (0.80, 1.34)	5 (0.4)	0.88 (0.27, 2.89)	0.78 (0.21, 2.97)
Campylobacter jejuni/coli	0	1715	1507 (87.9)	1.	1.	54 (3.1)	1.	1.
	1	1507	1288 (85.5)	1.60 (1.47, 1.75)	1.61 (1.46, 1.77)	42 (2.8)	1.52 (0.97, 2.39)	1.57 (0.97, 2.55)
	2+	1288	1071 (83.2)	1.89 (1.74, 2.06)	1.81 (1.64, 1.99)	52 (4.0)	1.30 (0.79, 2.15)	1.31 (0.79, 2.18)
Cryptosporidium	0	1715	1236 (72.1)	1.	1.	67 (3.9)	1.	1.
	1	1236	819 (66.3)	1.71 (1.53, 1.92)	1.56 (1.26, 1.91)	27 (2.2)	0.83 (0.52, 1.33)	0.68 (0.42, 1.11)
	2+	819	484 (59.1)	1.72 (1.52, 1.95)	1.47 (1.06, 2.05)	17 (2.1)	0.36 (0.20, 0.64)	0.27 (0.15, 0.49)

*Hazard ratio adjusted for site, SES, sex, enrollment WAZ, maternal education, maternal height, crowding, and exclusive breastfeeding in first 6 months

†Hazard ratio adjusted for the same variables above and calibrated based on negative control estimates

Table S7. Estimates of protection with minimum duration between new infections at 14 days instead of 21.

			Infection outcome			Attributable diarrhea outcome		
Pathogen	# previous infections	N	N (%) with subsequent infection	HR* (95% CI)	Calibrated† HR (95% CI)	N (%) with subsequent diarrhea	HR* (95% CI)	Calibrated† HR (95% CI)
Rotavirus	0	1715	839 (48.9)	1.	1.	374 (21.8)	1.	1.
	1	839	284 (33.8)	0.86 (0.74, 1.01)	0.81 (0.66, 0.99)	122 (14.5)	0.62 (0.50, 0.78)	0.53 (0.42, 0.66)
	2+	284	84 (29.6)	0.87 (0.67, 1.12)	0.79 (0.60, 1.02)	50 (17.6)	0.63 (0.47, 0.85)	0.48 (0.35, 0.67)
Astrovirus	0	1715	1048 (61.1)	1.	1.	174 (10.1)	1.	1.
	1	1048	492 (46.9)	0.85 (0.76, 0.96)	0.81 (0.67, 0.98)	83 (7.9)	0.75 (0.57, 0.99)	0.66 (0.50, 0.87)
	2+	492	181 (36.8)	0.78 (0.66, 0.92)	0.77 (0.64, 0.93)	45 (9.1)	0.71 (0.49, 1.03)	0.58 (0.40, 0.84)
Norovirus GII	0	1715	1310 (76.4)	1.	1.	138 (8.0)	1.	1.
	1	1310	812 (62.0)	1.02 (0.92, 1.13)	1.02 (0.91, 1.13)	90 (6.9)	0.79 (0.59, 1.05)	0.75 (0.53, 1.06)
	2+	812	397 (48.9)	0.86 (0.76, 0.97)	0.91 (0.80, 1.03)	63 (7.8)	0.56 (0.40, 0.77)	0.55 (0.39, 0.76)
Sapovirus	0	1715	1350 (78.7)	1.	1.	227 (13.2)	1.	1.
	1	1350	841 (62.3)	0.96 (0.87, 1.06)	0.95 (0.85, 1.06)	149 (11.0)	0.88 (0.71, 1.10)	0.79 (0.63, 0.98)
	2+	841	425 (50.5)	0.88 (0.78, 0.98)	0.82 (0.73, 0.93)	130 (15.5)	1.08 (0.86, 1.37)	0.89 (0.69, 1.13)
Adenovirus 40/41	0	1715	1161 (67.7)	1.	1.	164 (9.6)	1.	1.
	1	1161	598 (51.5)	0.99 (0.88, 1.10)	0.91 (0.80, 1.03)	103 (8.9)	0.98 (0.75, 1.29)	0.83 (0.62, 1.11)
	2+	598	294 (49.2)	1.08 (0.93, 1.25)	1.01 (0.85, 1.19)	102 (17.1)	1.20 (0.89, 1.62)	0.92 (0.67, 1.27)
Shigella	0	1715	1141 (66.5)	1.	1.	386 (22.5)	1.	1.
	1	1141	729 (63.9)	1.76 (1.57, 1.97)	1.52 (1.26, 1.84)	170 (14.9)	1.11 (0.91, 1.35)	0.88 (0.72, 1.08)
	2+	729	428 (58.7)	1.80 (1.60, 2.02)	1.44 (1.01, 2.07)	138 (18.9)	0.99 (0.79, 1.23)	0.69 (0.55, 0.87)
ST-ETEC	0	1715	1244 (72.5)	1.	1.	136 (7.9)	1.	1.
	1	1244	831 (66.8)	1.07 (0.98, 1.18)	1.04 (0.94, 1.16)	143 (11.5)	1.48 (1.16, 1.89)	1.27 (0.98, 1.65)
	2+	831	518 (62.3)	1.06 (0.95, 1.18)	1.02 (0.91, 1.14)	118 (14.2)	1.10 (0.82, 1.47)	0.84 (0.62, 1.14)
tEPEC	0	1715	1251 (72.9)	1.	1.	23 (1.3)	1.	1.
	1	1251	840 (67.1)	1.25 (1.13, 1.39)	1.20 (0.98, 1.46)	6 (0.5)	1.00 (0.35, 2.86)	0.61 (0.21, 1.79)
	2+	840	465 (55.4)	1.01 (0.90, 1.13)	0.91 (0.69, 1.19)	2 (0.2)	0.47 (0.10, 2.15)	0.41 (0.08, 2.14)
Campylobacter jejuni/coli	0	1715	1191 (69.4)	1.	1.	70 (4.1)	1.	1.
	1	1191	856 (71.9)	1.39 (1.25, 1.55)	1.40 (1.25, 1.56)	38 (3.2)	1.39 (0.88, 2.17)	1.43 (0.89, 2.31)
	2+	856	578 (67.5)	1.76 (1.58, 1.97)	1.68 (1.49, 1.90)	43 (5.0)	1.48 (0.92, 2.38)	1.49 (0.92, 2.42)
Cryptosporidium	0	1715	950 (55.4)	1.	1.	84 (4.9)	1.	1.
	1	950	362 (38.1)	0.98 (0.85, 1.14)	0.89 (0.71, 1.12)	19 (2.0)	0.53 (0.32, 0.89)	0.44 (0.26, 0.74)
	2+	362	126 (34.8)	0.94 (0.77, 1.14)	0.80 (0.56, 1.16)	12 (3.3)	0.46 (0.24, 0.87)	0.35 (0.18, 0.66)

*Hazard ratio adjusted for site, SES, sex, enrollment WAZ, maternal education, maternal height, crowding, and exclusive breastfeeding in first 6 months
†Hazard ratio adjusted for the same variables above and calibrated based on negative control estimates

Table S8. Estimates of protection with minimum duration between new infections at 31 days instead of 21.

			Infection outcome			Attributable diarrhea outcome		
Pathogen	# previous infections	N	N (%) with subsequent infection	HR* (95% CI)	Calibrated† HR (95% CI)	N (%) with subsequent diarrhea	HR* (95% CI)	Calibrated† HR (95% CI)
Rotavirus	0	1715	839 (48.9)	1.	1.	382 (22.3)	1.	1.
	1	839	248 (29.6)	0.70 (0.60, 0.82)	0.66 (0.53, 0.81)	125 (14.9)	0.59 (0.47, 0.73)	0.50 (0.40, 0.62)
	2+	248	64 (25.8)	0.79 (0.58, 1.07)	0.71 (0.52, 0.98)	35 (13.9)	0.51 (0.36, 0.72)	0.39 (0.27, 0.56)
Astrovirus	0	1715	1048 (61.1)	1.	1.	181 (10.6)	1.	1.
	1	1048	464 (44.3)	0.79 (0.70, 0.89)	0.75 (0.62, 0.90)	86 (8.2)	0.72 (0.55, 0.96)	0.63 (0.48, 0.84)
	2+	464	152 (32.8)	0.71 (0.60, 0.85)	0.70 (0.57, 0.86)	34 (7.3)	0.61 (0.41, 0.90)	0.49 (0.33, 0.74)
Norovirus GII	0	1715	1310 (76.4)	1.	1.	143 (8.3)	1.	1.
	1	1310	771 (58.9)	0.89 (0.81, 0.99)	0.89 (0.80, 0.99)	92 (7.0)	0.74 (0.55, 0.98)	0.70 (0.50, 0.99)
	2+	771	338 (43.8)	0.77 (0.68, 0.88)	0.81 (0.71, 0.93)	56 (7.3)	0.63 (0.45, 0.90)	0.62 (0.44, 0.89)
Sapovirus	0	1715	1350 (78.7)	1.	1.	232 (13.5)	1.	1.
	1	1350	808 (59.9)	0.88 (0.80, 0.97)	0.87 (0.77, 0.97)	164 (12.1)	0.93 (0.75, 1.15)	0.83 (0.67, 1.03)
	2+	808	358 (44.3)	0.76 (0.67, 0.86)	0.71 (0.62, 0.81)	104 (12.9)	1.03 (0.79, 1.35)	0.85 (0.64, 1.12)
Adenovirus 40/41	0	1715	1161 (67.7)	1.	1.	169 (9.9)	1.	1.
	1	1161	570 (49.1)	0.91 (0.81, 1.01)	0.84 (0.74, 0.95)	110 (9.5)	1.00 (0.77, 1.30)	0.85 (0.64, 1.12)
	2+	570	257 (45.1)	0.94 (0.80, 1.11)	0.88 (0.74, 1.06)	82 (14.4)	0.99 (0.72, 1.38)	0.76 (0.54, 1.07)
Shigella	0	1715	1141 (66.5)	1.	1.	396 (23.1)	1.	1.
	1	1141	663 (58.1)	1.38 (1.23, 1.54)	1.19 (0.98, 1.44)	180 (15.8)	1.01 (0.83, 1.22)	0.80 (0.65, 0.98)
	2+	663	332 (50.1)	1.34 (1.18, 1.52)	1.08 (0.75, 1.55)	104 (15.6)	0.93 (0.73, 1.18)	0.65 (0.51, 0.84)
ST-ETEC	0	1715	1244 (72.5)	1.	1.	143 (8.3)	1.	1.
	1	1244	815 (65.5)	1.00 (0.91, 1.10)	0.97 (0.87, 1.07)	147 (11.8)	1.33 (1.05, 1.70)	1.15 (0.88, 1.49)
	2+	815	495 (60.7)	0.95 (0.85, 1.06)	0.92 (0.81, 1.03)	106 (13.0)	0.92 (0.69, 1.23)	0.70 (0.52, 0.95)
tEPEC	0	1715	1251 (72.9)	1.	1.	25 (1.5)	1.	1.
	1	1251	807 (64.5)	1.05 (0.95, 1.17)	1.01 (0.83, 1.23)	4 (0.3)	0.54 (0.18, 1.66)	0.33 (0.11, 1.04)
	2+	807	390 (48.3)	0.82 (0.73, 0.93)	0.74 (0.57, 0.98)	2 (0.2)	0.57 (0.11, 2.83)	0.50 (0.09, 2.80)
Campylobacter jejuni/coli	0	1715	1191 (69.4)	1.	1.	74 (4.3)	1.	1.
	1	1191	821 (68.9)	1.20 (1.08, 1.33)	1.20 (1.07, 1.34)	41 (3.4)	1.36 (0.86, 2.16)	1.41 (0.86, 2.30)
	2+	821	515 (62.7)	1.41 (1.25, 1.58)	1.34 (1.18, 1.52)	35 (4.3)	1.45 (0.87, 2.42)	1.47 (0.87, 2.45)
Cryptosporidium	0	1715	950 (55.4)	1.	1.	87 (5.1)	1.	1.
	1	950	314 (33.1)	0.77 (0.67, 0.89)	0.70 (0.56, 0.88)	18 (1.9)	0.43 (0.26, 0.69)	0.35 (0.21, 0.58)
	2+	314	80 (25.5)	0.67 (0.52, 0.85)	0.57 (0.39, 0.84)	6 (1.9)	0.29 (0.12, 0.73)	0.22 (0.09, 0.55)

*Hazard ratio adjusted for site, SES, sex, enrollment WAZ, maternal education, maternal height, crowding, and exclusive breastfeeding in first 6 months

†Hazard ratio adjusted for the same variables above and calibrated based on negative control estimates

Table S9. Estimates of protection with prior attributable diarrhea instead of prior infection as the definition of prior exposure resulting in immunity.

			Infection outcome			Attributable diarrhea outcome		
Pathogen	# previous diarrhea	N	N (%) with subsequent infection	HR* (95% CI)	Calibrated† HR (95% CI)	N (%) with subsequent diarrhea	HR* (95% CI)	Calibrated† HR (95% CI)
Rotavirus	0	1715	839 (48.9)	1.	1.	446 (26.0)	1.	1.
	1	446	148 (33.2)	0.79 (0.66, 0.95)	0.74 (0.59, 0.93)	93 (20.9)	0.72 (0.57, 0.92)	0.61 (0.48, 0.78)
	2+	93	21 (22.6)	0.54 (0.33, 0.86)	0.49 (0.30, 0.78)	11 (11.8)	0.42 (0.23, 0.76)	0.32 (0.17, 0.59)
Astrovirus	0	1715	1048 (61.1)	1.	1.	261 (15.2)	1.	1.
	1	261	120 (46.0)	0.97 (0.82, 1.15)	0.92 (0.74, 1.15)	37 (14.2)	1.05 (0.73, 1.50)	0.92 (0.64, 1.32)
	2+	37	15 (40.5)	1.05 (0.66, 1.68)	1.03 (0.64, 1.67)	4 (10.8)	0.93 (0.35, 2.47)	0.75 (0.28, 2.01)
Norovirus GII	0	1715	1310 (76.4)	1.	1.	254 (14.8)	1.	1.
	1	254	137 (53.9)	1.15 (1.00, 1.33)	1.14 (0.99, 1.32)	36 (14.2)	0.89 (0.63, 1.25)	0.85 (0.57, 1.25)
	2+	36	15 (41.7)	1.06 (0.72, 1.54)	1.11 (0.76, 1.63)	2 (5.6)	0.56 (0.14, 2.29)	0.55 (0.13, 2.25)
Sapovirus	0	1715	1350 (78.7)	1.	1.	388 (22.6)	1.	1.
	1	388	242 (62.4)	1.06 (0.94, 1.20)	1.05 (0.91, 1.19)	112 (28.9)	1.51 (1.22, 1.88)	1.35 (1.08, 1.69)
	2+	112	57 (50.9)	1.05 (0.84, 1.32)	0.99 (0.78, 1.24)	24 (21.4)	1.40 (0.95, 2.08)	1.15 (0.77, 1.72)
Adenovirus 40/41	0	1715	1161 (67.7)	1.	1.	247 (14.4)	1.	1.
	1	247	170 (68.8)	1.09 (0.92, 1.28)	1.00 (0.84, 1.19)	90 (36.4)	1.22 (0.93, 1.60)	1.03 (0.77, 1.38)
	2+	90	55 (61.1)	0.98 (0.78, 1.24)	0.92 (0.72, 1.17)	38 (42.2)	1.35 (0.94, 1.95)	1.04 (0.72, 1.52)
Shigella	0	1715	1141 (66.5)	1.	1.	507 (29.6)	1.	1.
	1	507	324 (63.9)	1.46 (1.29, 1.65)	1.26 (1.03, 1.53)	160 (31.6)	1.18 (0.97, 1.44)	0.94 (0.76, 1.15)
	2+	160	86 (53.8)	1.38 (1.13, 1.68)	1.11 (0.75, 1.65)	43 (26.9)	1.16 (0.85, 1.58)	0.81 (0.59, 1.12)
ST-ETEC	0	1715	1244 (72.5)	1.	1.	330 (19.2)	1.	1.
	1	330	223 (67.6)	1.07 (0.96, 1.19)	1.04 (0.93, 1.17)	82 (24.8)	1.27 (0.97, 1.65)	1.09 (0.82, 1.45)
	2+	82	52 (63.4)	1.12 (0.90, 1.38)	1.07 (0.86, 1.33)	21 (25.6)	1.52 (0.94, 2.47)	1.16 (0.71, 1.90)
tEPEC	0	1715	1251 (72.9)	1.	1.	29 (1.7)	1.	1.
	1	29	18 (62.1)	0.83 (0.60, 1.14)	0.79 (0.55, 1.14)	2 (6.9)	5.58 (1.44, 21.68)	3.41 (0.86, 13.48)
	2+	2	2 (100.0)	0.71 (0.34, 1.51)	0.64 (0.29, 1.42)	0	—	—
Campylobacter jejuni/coli	0	1715	1191 (69.4)	1.	1.	124 (7.2)	1.	1.
	1	124	86 (69.4)	1.17 (0.96, 1.44)	1.17 (0.95, 1.44)	25 (20.2)	1.29 (0.79, 2.11)	1.34 (0.80, 2.24)
	2+	25	14 (56.0)	1.01 (0.60, 1.72)	0.97 (0.57, 1.64)	6 (24.0)	1.79 (0.80, 3.99)	1.80 (0.80, 4.05)
Cryptosporidium	0	1715	950 (55.4)	1.	1.	102 (5.9)	1.	1.
	1	102	48 (47.1)	1.28 (1.00, 1.63)	1.16 (0.86, 1.57)	9 (8.8)	1.35 (0.71, 2.59)	1.11 (0.57, 2.15)
	2+	9	1 (11.1)	0.60 (0.06, 6.16)	0.51 (0.05, 5.39)	0	—	—

*Hazard ratio adjusted for site, SES, sex, enrollment WAZ, maternal education, maternal height, crowding, and exclusive breastfeeding in first 6 months

†Hazard ratio adjusted for the same variables above and calibrated based on negative control estimates

Table S10. Estimates of protection excluding diarrhea episodes with multiple attributable pathogens.

Attributable diarrhea outcome					
Pathogen	# previous infections	N	N (%) with subsequent diarrhea	HR* (95% CI)	Calibrated† HR (95% CI)
Rotavirus	0	1715	298 (17.4)	1.	1.
	1	839	87 (10.4)	0.57 (0.44, 0.74)	0.48 (0.37, 0.63)
	2+	271	22 (8.1)	0.39 (0.25, 0.61)	0.30 (0.19, 0.47)
Astrovirus	0	1715	134 (7.8)	1.	1.
	1	1048	54 (5.2)	0.69 (0.49, 0.97)	0.60 (0.43, 0.85)
	2+	483	29 (6.0)	0.73 (0.47, 1.16)	0.60 (0.38, 0.95)
Norovirus GII	0	1715	116 (6.8)	1.	1.
	1	1310	59 (4.5)	0.58 (0.41, 0.81)	0.55 (0.37, 0.81)
	2+	799	36 (4.5)	0.41 (0.27, 0.61)	0.40 (0.26, 0.60)
Sapovirus	0	1715	159 (9.3)	1.	1.
	1	1350	94 (7.0)	0.84 (0.64, 1.10)	0.75 (0.57, 0.99)
	2+	836	80 (9.6)	1.08 (0.79, 1.48)	0.88 (0.64, 1.22)
Adenovirus 40/41	0	1715	98 (5.7)	1.	1.
	1	1161	60 (5.2)	1.28 (0.90, 1.84)	1.09 (0.75, 1.58)
	2+	591	42 (7.1)	1.17 (0.74, 1.84)	0.90 (0.57, 1.43)
Shigella	0	1715	256 (14.9)	1.	1.
	1	1141	111 (9.7)	1.14 (0.89, 1.46)	0.90 (0.70, 1.16)
	2+	720	79 (11.0)	0.99 (0.74, 1.32)	0.69 (0.52, 0.93)
ST-ETEC	0	1715	82 (4.8)	1.	1.
	1	1244	69 (5.5)	1.21 (0.86, 1.69)	1.04 (0.73, 1.48)
	2+	825	62 (7.5)	0.97 (0.65, 1.46)	0.74 (0.49, 1.13)
tEPEC	0	1715	16 (0.9)	1.	1.
	1	1251	5 (0.4)	1.56 (0.50, 4.86)	0.95 (0.30, 3.03)
	2+	835	1 (0.1)	0.68 (0.09, 5.38)	0.60 (0.07, 5.20)
Campylobacter jejuni/coli	0	1715	46 (2.7)	1.	1.
	1	1191	21 (1.8)	1.45 (0.81, 2.59)	1.50 (0.82, 2.74)
	2+	851	23 (2.7)	1.68 (0.86, 3.25)	1.69 (0.87, 3.29)
Cryptosporidium	0	1715	58 (3.4)	1.	1.
	1	950	11 (1.2)	0.39 (0.20, 0.74)	0.32 (0.16, 0.61)
	2+	346	6 (1.7)	0.33 (0.14, 0.76)	0.25 (0.11, 0.58)

*Hazard ratio adjusted for site, SES, sex, enrollment WAZ, maternal education, maternal height, crowding, and exclusive breastfeeding in first 6 months

†Hazard ratio adjusted for the same variables above and calibrated based on negative control estimates

Table S11. Estimates of protection comparing calibration with negative controls of the same pathogen type (primary analysis) and with all pathogens (sensitivity analysis).

		Infection outcome				Attributable diarrhea outcome			
Pathogen	# previous infections	Mean (SD) bias	Calibrated* HR (95% CI)	Mean (SD) bias	All pathogen calibrated† HR (95% CI)	Mean (SD) bias	Calibrated* HR (95% CI)	Mean (SD) bias	All pathogen calibrated† HR (95% CI)
Rotavirus	0		1.		1.		1.		1
	1	0.06 (0.07)	0.74 (0.61, 0.91)	0.00 (0.05)	0.79 (0.66, 0.95)	0.17 (0.02)	0.53 (0.42, 0.66)	0.05 (0.06)	0.59 (0.46, 0.77)
	2+	0.10 (0.03)	0.73 (0.55, 0.97)	-0.01 (0.06)	0.81 (0.60, 1.10)	0.26 (0.05)	0.43 (0.31, 0.61)	0.14 (0.42)	0.49 (0.20, 1.18)
Astrovirus	0		1.		1.		1.		1
	1	0.05 (0.07)	0.78 (0.65, 0.94)	-0.01 (0.06)	0.83 (0.70, 0.98)	0.13 (0.04)	0.66 (0.49, 0.87)	0.04 (0.05)	0.72 (0.54, 0.96)
	2+	0.02 (0.05)	0.72 (0.60, 0.88)	-0.05 (0.08)	0.78 (0.62, 0.97)	0.21 (0.04)	0.52 (0.35, 0.77)	0.11 (0.07)	0.57 (0.38, 0.86)
Norovirus GII	0		1.		1.		1.		1
	1	0.01 (0.02)	0.96 (0.86, 1.07)	-0.04 (0.03)	1.01 (0.90, 1.13)	0.05 (0.10)	0.73 (0.52, 1.03)	-0.05 (0.06)	0.81 (0.59, 1.10)
	2+	-0.05 (0.02)	0.88 (0.78, 1.01)	-0.13 (0.08)	0.96 (0.79, 1.17)	0.02 (0.04)	0.56 (0.40, 0.79)	-0.08 (0.03)	0.62 (0.44, 0.87)
Sapovirus	0		1.		1.		1.		1
	1	0.02 (0.03)	0.92 (0.82, 1.03)	-0.01 (0.03)	0.94 (0.84, 1.06)	0.11 (0.02)	0.82 (0.66, 1.02)	0.03 (0.03)	0.89 (0.72, 1.11)
	2+	0.07 (0.02)	0.78 (0.69, 0.88)	-0.03 (0.05)	0.86 (0.73, 1.00)	0.20 (0.03)	0.84 (0.65, 1.09)	0.08 (0.04)	0.95 (0.73, 1.23)
Adenovirus 40/41	0		1.		1.		1.		1
	1	0.08 (0.03)	0.89 (0.78, 1.01)	0.02 (0.03)	0.94 (0.83, 1.06)	0.17 (0.05)	0.87 (0.65, 1.16)	0.13 (0.03)	0.90 (0.69, 1.19)
	2+	0.07 (0.03)	0.95 (0.81, 1.13)	-0.04 (0.09)	1.06 (0.84, 1.34)	0.26 (0.05)	0.82 (0.59, 1.14)	0.14 (0.12)	0.93 (0.63, 1.37)
Shigella	0		1.		1.		1.		1
	1	0.15 (0.08)	1.46 (1.21, 1.77)	0.12 (0.08)	1.50 (1.24, 1.81)	0.23 (0.02)	0.85 (0.69, 1.04)	0.18 (0.02)	0.89 (0.73, 1.09)
	2+	0.22 (0.17)	1.37 (0.95, 1.96)	0.14 (0.16)	1.48 (1.06, 2.06)	0.35 (0.03)	0.67 (0.53, 0.84)	0.23 (0.03)	0.76 (0.60, 0.95)
ST-ETEC	0		1.		1.		1.		1
	1	0.03 (0.02)	1.02 (0.92, 1.13)	0.02 (0.01)	1.03 (0.93, 1.14)	0.15 (0.05)	1.25 (0.96, 1.63)	0.19 (0.10)	1.20 (0.88, 1.65)
	2+	0.04 (0.02)	1.00 (0.90, 1.13)	0.02 (0.01)	1.02 (0.91, 1.14)	0.27 (0.05)	0.84 (0.62, 1.13)	0.23 (0.06)	0.87 (0.64, 1.18)
tEPEC	0		1.		1.		1.		1
	1	0.05 (0.09)	1.15 (0.94, 1.40)	0.04 (0.08)	1.16 (0.95, 1.40)	0.49 (0.12)	0.60 (0.21, 1.75)	0.36 (0.06)	0.69 (0.24, 1.96)
	2+	0.10 (0.13)	0.86 (0.66, 1.13)	0.05 (0.08)	0.91 (0.75, 1.11)	0.12 (0.31)	0.46 (0.09, 2.48)	0.13 (0.12)	0.46 (0.10, 2.24)
Campylobacter jejuni/coli	0		1.		1.		1.		1
	1	0.00 (0.02)	1.35 (1.20, 1.51)	-0.01 (0.02)	1.35 (1.21, 1.51)	-0.03 (0.09)	1.34 (0.82, 2.16)	0.01 (0.04)	1.28 (0.81, 2.03)
	2+	0.05 (0.02)	1.63 (1.45, 1.84)	0.05 (0.02)	1.62 (1.44, 1.83)	-0.01 (0.04)	1.43 (0.88, 2.34)	0.05 (0.03)	1.35 (0.83, 2.19)
Cryptosporidium	0		1.		1.		1.		1
	1	0.10 (0.09)	0.81 (0.65, 1.02)	0.10 (0.05)	0.81 (0.68, 0.97)	0.20 (0.06)	0.35 (0.21, 0.59)	0.15 (0.05)	0.36 (0.22, 0.61)
	2+	0.15 (0.15)	0.71 (0.49, 1.03)	0.16 (0.11)	0.71 (0.53, 0.95)	0.28 (0.05)	0.25 (0.11, 0.53)	0.28 (0.06)	0.25 (0.11, 0.54)

*Hazard ratio adjusted for site, SES, sex, enrollment WAZ, maternal education, maternal height, crowding, and exclusive breastfeeding in first 6 months and calibrated based on negative controls of the same pathogen type (bacteria, viruses, and parasites).
†Hazard ratio adjusted for the same variables and calibrated based on negative controls of all pathogens.

Table S12. Estimates of protection comparing adjustment for pre-specified confounders (primary analysis), adjustment for pre-specified confounders and prior exposure to other pathogens (sensitivity analysis), and calibration with negative controls (primary analysis).

			Infection outcome				Attributable diarrhea outcome			
Pathogen	# previous infections	N	N (%) with subsequent infection	Primary analysis adjusted* HR (95% CI)	Other pathogen adjusted† HR (95% CI)	Calibrated‡ HR (95% CI)	N (%) with subsequent diarrhea	Primary analysis adjusted* HR (95% CI)	Other pathogen adjusted† HR (95% CI)	Calibrated‡ HR (95% CI)
Rotavirus	0	1715	839 (48.9)	1.	1.	1.	375 (21.9)	1.	1.	1.
	1	839	271 (32.3)	0.79 (0.68, 0.93)	0.77 (0.66, 0.90)	0.74 (0.61, 0.91)	127 (15.1)	0.63 (0.50, 0.78)	0.59 (0.47, 0.73)	0.53 (0.42, 0.66)
	2+	271	75 (27.7)	0.81 (0.61, 1.07)	0.76 (0.57, 1.01)	0.73 (0.55, 0.97)	42 (15.5)	0.56 (0.40, 0.78)	0.49 (0.36, 0.68)	0.43 (0.31, 0.61)
Astrovirus	0	1715	1048 (61.1)	1.	1.	1.	177 (10.3)	1.	1.	1.
	1	1048	482 (46.0)	0.82 (0.73, 0.92)	0.81 (0.72, 0.91)	0.78 (0.65, 0.94)	85 (8.1)	0.75 (0.57, 0.98)	0.71 (0.54, 0.93)	0.66 (0.49, 0.87)
	2+	482	173 (35.9)	0.74 (0.62, 0.87)	0.72 (0.61, 0.86)	0.72 (0.60, 0.88)	39 (8.1)	0.64 (0.43, 0.94)	0.59 (0.39, 0.88)	0.52 (0.35, 0.77)
Norovirus GII	0	1715	1310 (76.4)	1.	1.	1.	140 (8.2)	1.	1.	1.
	1	1310	799 (61.0)	0.96 (0.87, 1.07)	0.96 (0.87, 1.07)	0.96 (0.86, 1.07)	91 (6.9)	0.77 (0.57, 1.02)	0.76 (0.57, 1.02)	0.73 (0.52, 1.03)
	2+	799	386 (48.3)	0.84 (0.74, 0.95)	0.84 (0.74, 0.95)	0.88 (0.78, 1.01)	60 (7.5)	0.57 (0.41, 0.80)	0.57 (0.40, 0.80)	0.56 (0.40, 0.79)
Sapovirus	0	1715	1350 (78.7)	1.	1.	1.	229 (13.4)	1.	1.	1.
	1	1350	833 (61.7)	0.93 (0.85, 1.03)	0.93 (0.84, 1.02)	0.92 (0.82, 1.03)	158 (11.7)	0.92 (0.75, 1.14)	0.90 (0.72, 1.11)	0.82 (0.66, 1.02)
	2+	833	405 (48.6)	0.83 (0.74, 0.94)	0.81 (0.72, 0.91)	0.78 (0.69, 0.88)	120 (14.4)	1.03 (0.80, 1.32)	0.98 (0.76, 1.26)	0.84 (0.65, 1.09)
Adenovirus 40/41	0	1715	1161 (67.7)	1.	1.	1.	166 (9.7)	1.	1.	1.
	1	1161	591 (50.9)	0.96 (0.86, 1.07)	0.95 (0.85, 1.07)	0.89 (0.78, 1.01)	109 (9.4)	1.03 (0.78, 1.34)	1.00 (0.76, 1.31)	0.87 (0.65, 1.16)
	2+	591	286 (48.4)	1.02 (0.87, 1.19)	0.98 (0.84, 1.15)	0.95 (0.81, 1.13)	92 (15.6)	1.07 (0.78, 1.46)	0.99 (0.72, 1.37)	0.82 (0.59, 1.14)
Shigella	0	1715	1141 (66.5)	1.	1.	1.	391 (22.8)	1.	1.	1.
	1	1141	719 (63.0)	1.69 (1.51, 1.90)	1.60 (1.43, 1.80)	1.46 (1.21, 1.77)	171 (15.0)	1.07 (0.87, 1.30)	0.98 (0.80, 1.21)	0.85 (0.69, 1.04)
	2+	719	405 (56.3)	1.70 (1.52, 1.91)	1.55 (1.37, 1.75)	1.37 (0.95, 1.96)	126 (17.5)	0.95 (0.76, 1.19)	0.82 (0.65, 1.04)	0.67 (0.53, 0.84)
ST-ETEC	0	1715	1244 (72.5)	1.	1.	1.	137 (8.0)	1.	1.	1.
	1	1244	825 (66.3)	1.05 (0.95, 1.16)	1.03 (0.94, 1.14)	1.02 (0.92, 1.13)	144 (11.6)	1.45 (1.13, 1.85)	1.36 (1.06, 1.74)	1.25 (0.96, 1.63)
	2+	825	513 (62.2)	1.04 (0.94, 1.16)	1.02 (0.92, 1.14)	1.00 (0.90, 1.13)	116 (14.1)	1.10 (0.82, 1.46)	1.02 (0.75, 1.37)	0.84 (0.62, 1.13)
tEPEC	0	1715	1251 (72.9)	1.	1.	1.	23 (1.3)	1.	1.	1.
	1	1251	835 (66.7)	1.21 (1.08, 1.34)	1.19 (1.07, 1.32)	1.15 (0.94, 1.40)	6 (0.5)	0.99 (0.35, 2.80)	0.84 (0.32, 2.18)	0.60 (0.21, 1.75)
	2+	835	452 (54.1)	0.96 (0.85, 1.07)	0.94 (0.84, 1.06)	0.86 (0.66, 1.13)	2 (0.2)	0.52 (0.11, 2.50)	0.36 (0.06, 1.99)	0.46 (0.09, 2.48)
Campylobacter jejuni/coli	0	1715	1191 (69.4)	1.	1.	1.	73 (4.3)	1.	1.	1.
	1	1191	850 (71.4)	1.35 (1.21, 1.50)	1.37 (1.23, 1.53)	1.35 (1.20, 1.51)	37 (3.1)	1.29 (0.82, 2.03)	1.32 (0.84, 2.08)	1.34 (0.82, 2.16)
	2+	850	569 (66.9)	1.71 (1.53, 1.91)	1.73 (1.54, 1.93)	1.63 (1.45, 1.84)	40 (4.7)	1.42 (0.87, 2.30)	1.55 (0.96, 2.50)	1.43 (0.88, 2.34)
Cryptosporidium	0	1715	950 (55.4)	1.	1.	1.	86 (5.0)	1.	1.	1.
	1	950	347 (36.5)	0.90 (0.78, 1.04)	0.86 (0.74, 1.00)	0.81 (0.65, 1.02)	17 (1.8)	0.42 (0.26, 0.70)	0.38 (0.22, 0.64)	0.35 (0.21, 0.59)
	2+	347	111 (32.0)	0.83 (0.68, 1.02)	0.79 (0.64, 0.97)	0.71 (0.49, 1.03)	8 (2.3)	0.32 (0.15, 0.70)	0.25 (0.12, 0.55)	0.25 (0.11, 0.53)

*Hazard ratio adjusted for site, SES, sex, enrollment WAZ, maternal education, maternal height, crowding, and exclusive breastfeeding in first 6 months; also shown in Figure 1A

†Hazard ratio adjusted for the same variables above and 1 and 2+ prior exposure to each other pathogen of the same type (bacteria, viruses, or parasites)

‡Hazard ratio adjusted for the same variables in * and calibrated based on negative control estimates; also shown in Figure 1B

References

1. Liu J, Gratz J, Amour C, et al. Optimization of Quantitative PCR Methods for Enteropathogen Detection. *PLoS ONE*. 2016;**11**(6):e0158199.

2. Liu J, Platts-Mills JA, Juma J, et al. Use of quantitative molecular diagnostic methods to identify causes of diarrhoea in children: a reanalysis of the GEMS case-control study. *Lancet*. 2016 Sep 24;**388**(10051):1291–1301.

3. Liu J, Gratz J, Amour C, et al. A Laboratory-Developed TaqMan Array Card for Simultaneous Detection of 19 Enteropathogens. *J Clin Microbiol*. 2013 Feb 1;**51**(2):472–480.

4. Liu J, Almeida M, Kabir F, et al. Direct Detection of Shigella in Stool Specimens by Use of a Metagenomic Approach. *J Clin Microbiol*. 2018 Feb;**56**(2).

5. Kelly PJ, Lim LL. Survival analysis for recurrent event data: an application to childhood infectious diseases. *Stat Med*. 2000 Jan 15;**19**(1):13–33.
